# Synthesis, DFT Analysis, and Evaluation of Antibacterial and Antioxidant Activities of Sulfathiazole Derivatives Combined with *In Silico* Molecular Docking and ADMET Predictions

**DOI:** 10.1155/2021/7534561

**Published:** 2021-12-14

**Authors:** Yoseph Samuel, Ankita Garg, Endale Mulugeta

**Affiliations:** Department of Applied Chemistry, School of Applied Natural Science, Adama Science and Technology University, P.O. Box 1888, Adama, Ethiopia

## Abstract

Synthetic modifications of sulfathiazole derivatives become an interesting approach to enhance their biological properties in line with their applications. As a result, sulfathiazole derivatives become a good candidate and potential class of organic compounds to play an important role towards medicinal chemistry. In present study, one thiazole derivative and two new sulfathiazole derivatives are synthesized with 94% and 72–81% yields, respectively. Furthermore, the synthesized compounds were evaluated for their *in vitro* antibacterial activity against two Gram-negative (*E. coli* and *P. aeruginosa*) and two Gram-positive bacterial strains (*S. pyogenes* and *S. aureus*) by disk diffusion method. Among synthesized compounds, compound **11a** showed potent inhibitory activity against Gram-negative, *E. coli* with 11.6 ± 0.283 mm zone of inhibition compared to standard drug sulfamethoxazole (15.7 ± 0.707 mm) at 50 mg/mL. The radical scavenging activities of these compounds were evaluated using DPPH radical assay, and compound **11a** showed the strongest activity with IC_50_ values of 1.655 *μ*g/mL. The synthesized compounds were evaluated for their *in silico* molecular docking analysis using *S. aureus* gyrase (PDB ID: 2XCT) and human myeloperoxidase (PDB ID: 1DNU) and were found to have minimum binding energy ranging from −7.8 to −10.0 kcal/mol with 2XCT and −7.5 to −9.7 with 1DNU. Compound **11a** showed very good binding score −9.7 kcal/mol with both of the proteins and had promising alignment with *in vitro* results. Compound 11b also showed high binding scores with both proteins. Drug likeness and ADMET of synthesized compounds were predicted. The DFT analysis of synthesized compounds was performed using Gaussian 09 and visualized through Gauss view 6.0. The structural coordinates of the lead compounds were optimized using B3LYP/6–31 G (d,p) level basis set without any symmetrical constraints. Studies revealed that all the synthesized compounds might be candidates for further antibacterial and antioxidant studies.

## 1. Introduction

Most of the organic compounds including natural products possess heterocyclic rings as a core part of it, and they provide the ability to alter their molecular conformation, solubility, physicochemical, pharmaceutical, and biological activities. These molecules perform remarkable functions in nature, medication, and innovation [1]. Heterocyclic compounds play an important role towards development of organic synthesis and have wide applications in the field of pharmaceutical science. Organic compounds with ring system containing sulfur, nitrogen, and oxygen as heteroatom are proven to be potent bioactive agents [2]. One of the most important groups of organic compounds among five-member heterocyclic compounds containing S and N atoms are called thiazoles and they belong to the group of azole heterocycles. Thiazole is structurally similar to imidazole and oxazole with the thiazole sulfur replaced by nitrogen in imidazole and oxygen in oxazole, respectively [3].

Thiazole derivatives act as antifungal [4], anti-inflammatory [5], analgesic [6], and anticancer agents [7]. On the other hand, sulfathiazole is an organic compound derived from sulfonamide by replacing one amide hydrogen atom with thiazole group. Sulfathiazole is the primary powerful bioactive agent efficiently utilized for the prevention and cure of bacterial infections.

**Figure 1:**
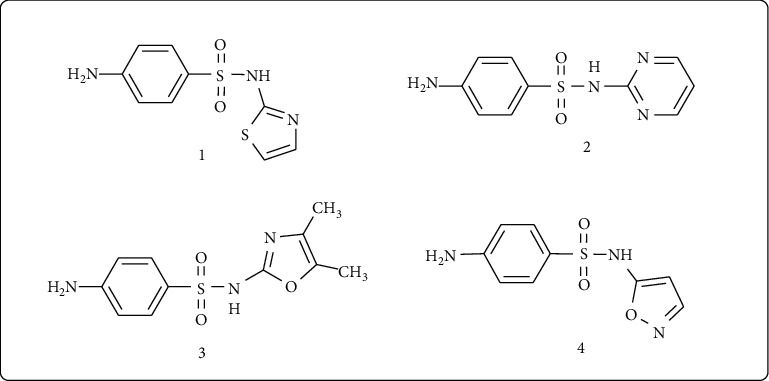
Chemical structure of few drugs containing sulfathiazole scaffold.

Sulfonamides, known as sulfa drugs, are the oldest drugs commonly employed and systematically used as bioactive agents. Few of these drugs containing sulfonamide moiety are sulfathiazole (**1**), sulfadiazine (**2**), sulfamoxole (**3**), and sulfafurazole (**4**) [8] (Figure 1). Sulfathiazole derivatives having heterocyclic scaffold possess wide applications for pharmaceutical purpose, such as antibacterial [9], antifungal [10], anti-inflammatory [8], and cytotoxic agents [11]. Nowadays, sulfathiazole bearing heterocyclic moieties have been synthesized and explored for their biological activities with specific target of diseases. Sulfathiazole bearing five member heterocyclic compounds have been widely studied due to their interesting applications as bioactive molecules [12]. After literature review of these traditional sulfonamides, we are reporting here synthesis of modified new derivatives to achieve sustainability in this area.

Drug development is a process in which we should strive to get novel drugs with optimum utilization of resources. We need a direction through pharmacokinetics and dynamics so that a lead can be decided, and we would not end up with clueless and baseless laboratory wastage. The pharmacological parameters such as drug likeness, ADME, and toxicity are providing promising insights in identifying lead compounds [13, 14]. In this process, molecular docking analysis and online ADMET predictions (SwissADME, Pro-Tox II and OSIRIS property explorer) are giving positive directions to researchers worldwide [15–17]. DFT analysis is helpful to optimize the geometry and identify the role of charge distribution to develop potential drug candidates [18–20]. DFT helps to get best binding mode during molecular docking studies as it minimizes the energy of the ligand and prepares it to get best fit within binding pocket of the enzyme. Synthesis and characterization of new sulfathiazole derivatives with detailed DFT study, as well as *in vitro* and *in silico* antibacterial and antioxidant analysis with pharmacological properties predictions such as drug likeness, ADME, and toxicity, are presented here for readers interest and benefit.

## 2. Materials and Methods

All solvents and chemicals were obtained commercially from fine chemicals PLC (Addis Ababa) and were used as received without further purification. Melting points were determined in an open capillary using digital melting point apparatus, expressed in ºC. Reaction progress was checked on precoated TLC plates and spots were visualized using UV light at 254 nm. Silica gel (60–120 mesh, Merck grade) has been used for column chromatography. The column was subjected to gradient elution by increasing ethyl acetate in *n*-hexane, and spots were visualized under UV lamp (254 nm). The synthesized compounds were characterized on the basis of physical and spectral analysis. The UV-Vis spectra of synthesized compounds were recorded on double-beam UV-Vis spectrophotometer using methanol as blank solvents for studying antioxidant activity. The ^1^H and ^13^C NMR spectra of the synthesized compounds were recorded on Bruker Avance 400 MHz NMR spectrophotometer using DMSO-*d*_*6*_ as the solvent, and the values are expressed in *δ* ppm.

### 2.1. Synthesis of 4-(4′-Nitrophenyl) Thiazol-2-Amine (7)

**Scheme 1:**
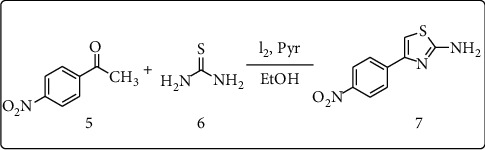
Synthesis of compound **7**.

4-(4′-Nitrophenyl) thiazol-2-amine **(7)** was synthesized using reported procedure developed by Abedi-Jazin et al. [21] (Scheme 1). Commercially available thiourea (3.04 g, 40 mmol), *p*-nitro acetophenone (3.3 g, 20 mmol), iodine (5.08 g, 20 mmol), and pyridine (2 drops) were mixed together and refluxed in ethanol (10 mL) at 100°C for 10 hr. The progress of the reaction was monitored using TLC in ethyl acetate/*n*-hexane (2 : 3) solvent system. After completion of the reaction, the mixture was cooled, extracted with diethyl ether to remove excess of acetophenone, and then washed with aqueous sodium thiosulfate to remove excess iodine and later with cold water. The crude product was dissolved in hot water and filtered to remove sulfonate, and the filtrate was basified with aqueous Na_2_CO_3_ to yield the corresponding 4-(4′-nitro phenyl) thiazol-2-amine (**7**). The crude product was purified by recrystallization from ethanol and provided high yield (94%). The spectroscopic and analytical data of compound are as follows:

Yellow powder, yield 94%, melting point 274–278 °C, R_f_ 0.68 (ethyl acetate/*n-*hexane, (2 : 3)).

^1^H NMR (400 MHz, DMSO-*d*_*6*_) *δ* 8.20 (2H, *s*, H-3**′**, H-5**′**), 8.01 (2H, *s*, H-2**′**, H-6**′**), 7.37 (1H, *s*, H-3). ^13^C NMR (100 MHz, DMSO-d_6_) *δ* 169.1 (C-2), 148.2 (C-4**′**), 146.3 (C-4), 141.2 (C-1**′**), 126.7 (C-2**′**, C-6**′**), 124.4 (C-3**′**, C-5**′**), and 107.1. DEPT-135 (100 MHz, DMSO-d_6_) *δ* 126.7 (C-2**′**, C-6**′**), 124.4 (C-3**′**, C-5**′**), and 107.1 (C-3).

### 2.2. Synthesis of Sulfathiazole Derivatives (11a-B)

**Scheme 2:**
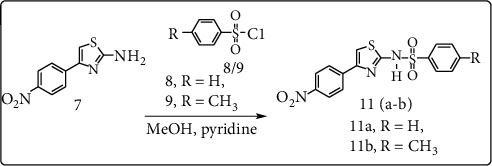
Synthesis of compounds **11** (**a-b**).

Sulfathiazole derivatives **(11a-b)** were synthesized according to protocol developed by Rehman et al., [22]. The intermediate 4-(4′-nitro phenyl) thiazol-2-amine **(7)** (4 mmol) was poured directly into 4 mmol of benzene/toluene sulfonyl chloride (**8/9)**, in the presence of pyridine (3 ml) in 40 mL of methanol for 24 hr at 25°C as represented in Scheme 2. The progress of the reaction was monitored using TLC in ethyl acetate/*n*-hexane (2 : 3) solvent system. After completion of the reaction, the mixture was poured into crushed ice, acidified by 10% hydrochloric acid, filtered, dried, and provided in good yield (72–81%). The spectroscopic and analytical data of compounds are as follows:

  4-(4′-nitrophenyl)-*N*-tosylthiazol-2-amine (11a):

Pale yellow powder, 81% yield, melting point 270–274°C, R_f_ value 0.64 ethyl acetate/*n-*hexane, (2 : 3). ^1^H NMR (400 MHz, DMSO *d*_*6*_) *δ* 7.85 (2H, d, *J* = 8.3 Hz, H-3**′**, H-5**′**), 7. 67 (2H, d, *J* = 6.9 Hz, H-2**″**, H-6**″**), 7.52 (2H, d, *J* = 8.4 Hz, H-2**′**, H-6**′**), 7.23 (2H, d, *J* = 9.0 Hz, H-3**″**, H-5**″**), *δ* 9.59 (1H, s, NH (Amide proton), 6.48 (1H, s, H-3, thiazole proton), 2.39 (3H, s, CH_3_). ^13^C NMR (100 MHz, DMSO-d_6_) *δ* 168.2 (C-2), 154.1 (C-4**′**), 151.2 (C-4), 148.4 (C-4**″**), 131.1 (C-1**′**), 127.1 (C-1**″**), 127.0 (C-3**″**, C-5**″**), 123.8 (C-2**′**, C-6**′**), 114.1 (C-2**″**, C-6**″**), 112.9 (C-3**′**, C-5**′**), 97.2 (C-3), and 26.3 (CH_3_). DEPT-135 (100 MHz, DMSO-d_6_) *δ* 127.0 (C-3**″**, C-5**″**), 123.8 (C-2**′**, C-6**′**), 114.1 (C-2**″**, C-6**″**), 112.9 (C-3**′**, C-5**′**), 97.2 (C-3), and 26.3 (CH_3_).

  4-(4′-nitrophenyl)-*N*-benzenesulfonylthiazol-2-amine (11b):

Pale yellow powder, yield 72%, melting point 270–274^o^C, R_f_ value 0.60 ethyl acetate/*n-*hexane, (2 : 3). ^1^H NMR (400 MHz, DMSO-d_6_) *δ* 9.70 (1H, *s*, NH), 6.89 (1H, *s*, H-3), 7.94 (1H, d, *J* = 8.2 Hz, H-3**′**, H-5**′**), 7.80 (2H, d, *J* = 8.2 Hz, H-2**″**, H-6**″**), 7.69 (2H, d, *J* = 8.2 Hz, H-2**′**, H-6**′**), 7.59 (2H, m, *J* = 8.7 Hz, 3**″**, 5**″**), 7.27 (1H, m, *J* = 12 Hz, H-4**″**). ^13^C NMR (100 MHz, DMSO-d_6_) *δ* 169.1 (C-2), 147.6 (C-4**′**), 146.4 (C-4), 144.2 (C-1**″**), 140.9 (C-1**′**), 129.9 (C-4**″**), 128.6 (C-3**″**, C-5**″**), 126.7 (C-2**′**, C-6**′**), 124.4 (C-2**″**, C-6**″**), 123.6 (C-3**′**, C-5**′**), 107.1 (C-3). DEPT-135 (100 MHz, DMSO-d_6_) *δ* 128.6 (C-3**″**, C-5**″**), 126.7 (C-2**′**, C-6**′**), 124.4 (C-2**″**, C-6**″**), 123.6 (C-3**′**, C-5**′**), 107.1 (C-3).

### 2.3. Antibacterial Activity

The synthesized compounds were evaluated for their *in vitro* antibacterial activity against two Gram-negative (*E. coli* and *P. aeruginosa*) and two Gram-positive bacteria *(S. pyogenes* and *S. aureus*). The bacterial cultures were inoculated into the nutrient broth (inoculation medium) and incubated overnight at 37°C. Inoculated medium was added aseptically to the nutrient medium and mixed thoroughly to get a uniform distribution. A solution of approximately 20 mL of sterile MHA was poured in sterile culture plates and allowed to attain room temperature. Sterile agar-disc diffusion previously soaked in a known concentration (50 mg/100 *μ*L, 25 mg/100 *μ*L, and 12.5 mg/100 *μ*L) of synthesized compounds and standard drug sulfamethoxazole were prepared in DMSO using nutrient agar tubes and carefully placed at the center of the labelled seeded plate. The zones of growth inhibition around the disks were measured after 24 hours of incubation at 37°C. The inhibition zones were measured with a ruler and compared with the positive control disk (disk containing sulfamethoxazole) and expressed in millimeter [23].

### 2.4. Antioxidant Activities of Sulfathiazole Derivatives

The free radical scavenging activities of the synthesized compound were measured by 1,1-diphenyl-2-picryl-hydrazyl (DPPH) method. With this method, it is possible to determine the radical scavenging power of an antioxidant by measuring the decrease in the absorbance of DPPH at 517 nm. As a result of the color changing from purple to yellow, the absorbance was decreased when the DPPH radical is scavenged by an antioxidant through donation of hydrogen to form a stable DPPH molecule. Lower absorbance of the reaction mixture indicated higher free radical scavenging activity [1].

### 2.5. *In Silico* Molecular Docking Methodology

#### 2.5.1. Preparation of Ligands

The 2D structures (.mol) of synthesized compounds (**7, 11a-b**) were drawn, and each individual structure was analyzed by using ChemDraw 16.0. The selected molecules were treated quantum mechanically by applying DFT method using the Gaussian 09 program suite at the Becke-3-Lee-YangPar (B3LYP) level combined with the standard 6-31G (d,p) basis set. During the optimization procedure, all the parameters were set in order to obtain a stable structure with minimum energy. The global minimum energy of the title compound was determined from the structure optimization procedure. The 3D coordinates (.PDB) of each molecule were obtained through optimized structure.

#### 2.5.2. Preparation of Macromolecules

The crystal structure of receptor molecules *S. aureus* gyrase (PDB ID: 2XCT) and human myeloperoxidase (PDB ID: 1DNU) were downloaded from protein data bank. As per standard protocol and practice worldwide, protein preparation was done. Water molecules and cofactors were selected to eliminate. Previously attached ligands were detached, and protein was prepared by adding polar hydrogens using auto preparation of target protein file AutoDock 4.2.6 (MGL tools 1.5.6).

#### 2.5.3. AutoDock Vina Analysis

The graphical user interface program AutoDock 4.2.6 was used to set the grid box for docking simulations. We tried several different docking pockets and poses, and finally the grid was generated as per best results achieved. The docking algorithm provided with AutoDock Vina was used to search for the best docked conformation between ligand and protein. A maximum of nine conformers were generated for each ligand. The conformations with the most favorable (least) free binding energy were selected for analyzing the interactions between the target protein and ligands by Discovery Studio Visualizer and PyMOL.

Auto Dock Vina with standard protocol was used to dock the protein *S. aureus* gyrase (PDB ID: 2XCT) and human myeloperoxidase (PDB ID: 1DNU) and synthesized ligands (**7, 11a-b**) into the active site of proteins. The molecular docking studies were carried out using AutoDock Tools (ADT) [17], which is a free graphic user interface (GUI) for the AutoDock Vina program. The grid box was constructed using 20 × 20 × 20, pointing in *x-*, y-, and *z-*directions, respectively, with a grid point spacing of 0.375 Å. The center grid box was of 62 × 30 × 62 Å for 2XCT and of 65 × 40 × 65 Å for 1DNU. Nine different conformations were generated for each ligand scored using AutoDock Vina functions and were ranked according to their binding energies. Binding pockets, H-bonds, and other hydrophobic and electrostatic interactions are shown by using different colours, sticks, ribbons, and lines.

### 2.6. *In Silico* Drug Likeness and Toxicity Predictions

This prediction directs users in the direction of drug efficiency and provides insights that studied ligand has properties consistent with being an orally active drug or not. This prediction is based on an already established concept by Lipinski et al., called Lipinski's rule of five [15]. The chemical structure of the compounds (**7, 11a-b**) was converted to their canonical simplified molecular input line entry system (SMILE) and submitted to SwissADME tool to estimate *in silico* pharmacokinetic parameters. SwissADME predictor provides information on the number of hydrogen donors, hydrogen acceptors and rotatable bonds, and total polar surface area of a compound. The ligands were also subjected to Lipinski et al., screened using SwissADME and PreADMET predictors. The organ toxicities and toxicological endpoints of the ligands and their LD_50_ were predicted using Pro Tox II and OSIRIS Property Explorer [15, 16]. The analyses of the compounds were compared with that of sulfathiazole and ascorbic acid standard drugs.

### 2.7. Quantum Computational Studies

The DFT (density functional theory) analysis of synthesized compounds was performed using Gaussian 09 and visualized through Gauss view 6.0. The structural coordinates of the lead compounds were optimized using B3LYP/6–31 G (d,p) level basis set without any symmetrical constraints. The molecular electrostatic potential map and energies of the compounds were obtained from the optimized geometry. Koopman's approximation was used to estimate the HOMO-LUMO energy gap and related reactive parameters (electronegativity, chemical potential, hardness, softness, and electrophilicity) [24, 25].

### 2.8. Statistical Data Analysis

The antimicrobial analysis data generated by triplicate measurements were reported as mean ± standard deviation. GraphPad Prism version 5.00 for Windows was used to perform the analysis (GraphPad Software, San Diego, California, USA, https://www.graphpad.com). Groups were analyzed for significant differences using a linear model of variance analysis (ANOVA) test, with significance accepted for *p* < 0.05.

## 3. Results and Discussion

### 3.1. Synthesis

In the present work, 4-(4′-nitrophenyl) thiazol-2-amine (**7**), 4-(4′-nitrophenyl)-*N*-tosylthiazol-2-amine (**11a**), and 4-(4′-nitrophenyl)-*N*-benzenesulfonylthiazol-2-amine (**11b**) were synthesized by the application of cyclization reaction and electrophilic substitution reaction. Structures of the synthesized compounds were confirmed based on the TLC, melting point, and NMR. 4-(4′-nitrophenyl) thiazol-2-amine (**7**) was synthesized by cyclization reaction between *p*-nitro acetophenone (5) and thiourea (6) [21]. Commercially available *p*-nitro acetophenone, iodine, thiourea, and 2 drops of pyridine were mixed together and refluxed in ethanol for 10 hrs to afford compound **7** in 94%.

4-(4′-Nitrophenyl)-*N*-tosylthiazol-2-amine (**11a**) and 4-(4′-nitrophenyl)-*N*-benzenesulfonylthiazol-2-amine (**11b**) were synthesized by electrophilic substitution reaction [22]. Compound **7** was reacted with benzene sulfonyl chloride/toluene sulfonyl chloride in methanol and the mixture was basified by dry pyridine to afford compound **11a**, **11b** with **81%,** and **72%** yield, respectively [26].

**Scheme 3:**
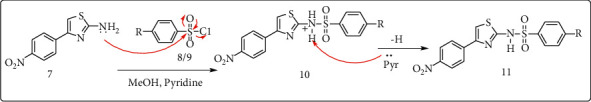
Proposed mechanism for synthesis of compounds **11** (**a-b**).

The plausible reaction mechanism of the compounds **11 (a-b)** starts with nucleophilic substitution reaction of amino thiazole with benzene sulfonyl chloride/toluene sulfonyl chloride as depicted in Scheme 3.

### 3.2. Antibacterial Activity of Synthesized Compounds

**Figure 2:**
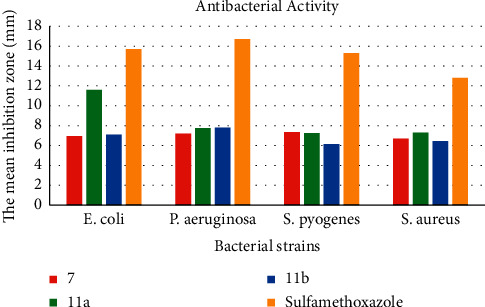
Mean inhibition zone of synthesized compounds in mm (mean ± SD) at 50 mg/mL.

The *in vitro* antibacterial activities of synthesized compounds were done against two Gram-negative (*E. coli* and *P. aeruginosa)* and two Gram-positive bacterial strains (*S. pyogenes* and *S. aureus*) by disk diffusion assay (Table 1). The results showed that all the tested compounds displayed potent to moderate antibacterial activity with inhibition zone of 6.00 ± 0.011 to 11.6 ± 0.283 mm (Figure 2). Compound **11a** displayed potent inhibitory activity with inhibition zone of 11.6 ± 0.283 mm. Comparing with compound **11b**, compound **11a** showed highest inhibitory activities; however, the difference between the structures of the two compounds differs only by methyl group attached to the benzene sulfonamide. From these results, it can be assumed that antibacterial activities of synthesized compounds increase with the number of carbons attached to the benzene sulfonamide increases.

### 3.3. Antioxidant Activity of Synthesized Compounds

DPPH is a simple method and is used to determine the radical scavenging power of an antioxidant by measuring the decrease in the absorbance of DPPH at 517 nm. In the DPPH scavenging assay, synthesized compounds were investigated for their free radical scavenging activities via their reaction with the stable DPPH radicals. The reduction of the DPPH was followed via the decrease in absorbance at 517 nm. Synthesized compounds significantly reduced the DPPH. The DPPH radical scavenging activities of synthesized compounds **7, 11a,** and **11b** were found to be **82.37%, 94.05%,** and **78.85%**, respectively, at 10 *μ*g/ml (Table 2) and ascorbic acid was found to be **97.57%**. It was observed that the DPPH scavenging activity increased with increasing concentration of the samples in the assay. For the various concentrations, compound **11a** exhibited slightly highest percent inhibition of the DPPH with IC_50_ of **1.655** as compared to the other synthesized compounds. The positive control, ascorbic acid showed maximum scavenging effect at very high concentration with IC_50_ of **1.526**.

### 3.4. Molecular Docking Studies

To understand the binding mode of the ligands, all the synthesized compounds were subjected to molecular docking studied against selected proteins, namely, *S. aureus* gyrase (PDB ID: 2XCT) and human myeloperoxidase (PDB ID: 1DNU) using AutoDock Vina [17].

#### 3.4.1. Binding Mode of Analysis of Synthesized Compounds (7, 11a-B) Docked against *S. aureus* Gyrase (PDB ID: 2XCT)

**Figure 3:**
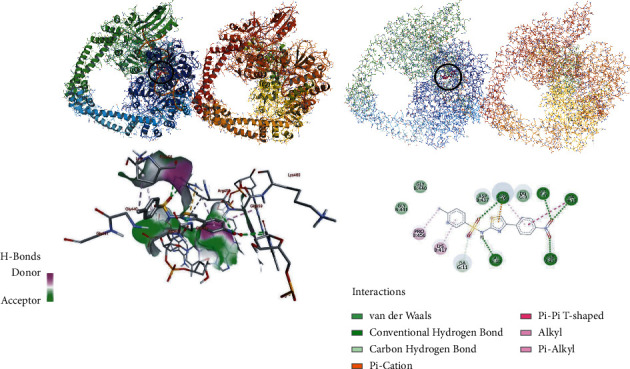
The 2D and 3D binding interactions of compound **11a** against *S. aureus* gyrase (PDB ID: 2XCT). 3D ribbon and line models show the binding pocket structure of *S. aureus* gyrase with compound **11a**. Hydrogen bonds between compounds and amino acids are shown as green dashed lines, and hydrophobic interactions are shown as pink lines. Electrostatic interaction is shown as orange line.

**Figure 4:**
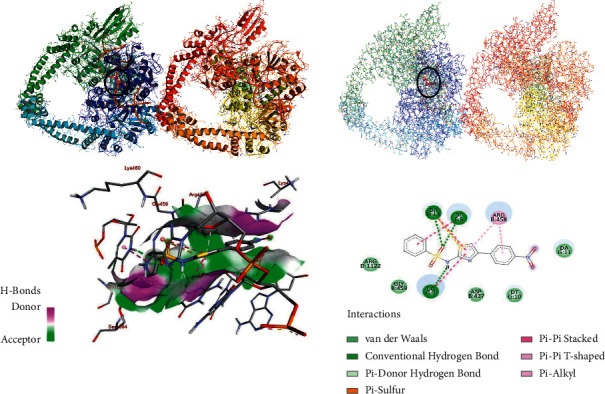
The 2D and 3D binding interactions of compound **11b** against *S. aureus* gyrase (PDB ID: 2XCT). 3D ribbon and line models show the binding pocket structure of *S. aureus* gyrase with compound **11b**. Hydrogen bonds between compounds and amino acids are shown as green dashed lines, and hydrophobic interactions are shown as pink/purple lines. Electrostatic interaction is shown as orange line.

Bacterial gyrase is paramount for bacterial survival and therefore necessary to disrupt as an antibacterial drug target [27]. Therefore, in this study, the molecular docking analysis of the synthesized compounds was carried out to investigate their binding pattern with bacterial gyrase and the results were compared with standard antibacterial agent sulfathiazole (see Supplementary Data**).** The synthesized compounds (**7, 11a-b**) were found to have minimum binding energy ranging from −7.8 to–10.0 kcal/mol (Table 3), with the best result achieved using compound **11a (– 9.7** kcal/mol) and **11b (–10.0** kcal/mol) (Figures 3 and 4). Comparing to sulfathiazole (– 7.4 kcal/mol), the synthesized compounds (**7, 11a-b**) have shown better binding affinity and similar residual interaction profile with amino acid residues. Hydrogen bonding interactions with various amino acids and bacterial DNA are also shown (Table 3). Based on the molecular docking analysis results, all the synthesized compounds have shown comparable residual interactions and docking scores with sulfathiazole. Therefore, these compounds might have potential to be promising antibacterial agents. The binding affinity, H-bond, and residual interaction of all the synthesized compounds are summarized in Table 3. The *in silico* results are in good agreement with *in vitro* results.

#### 3.4.2. Binding Mode of Analysis of Synthesized Compounds (7, 11a-B) Docked against Human Myeloperoxidase (PDB ID: 1DNU)

**Figure 5:**
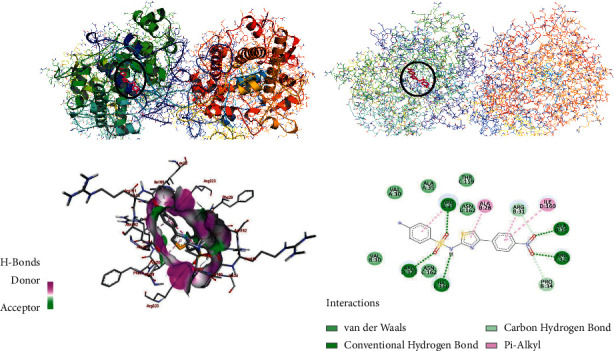
The 2D and 3D binding interactions of compound **11a** against human myeloperoxidase (PDB ID: 1DNU). 3D ribbon and line models show the binding pocket structure of human myeloperoxidase with compound **11a**. Hydrogen bonds between compounds and amino acids are shown as green dashed lines, and hydrophobic interactions are shown as pink lines.

**Figure 6:**
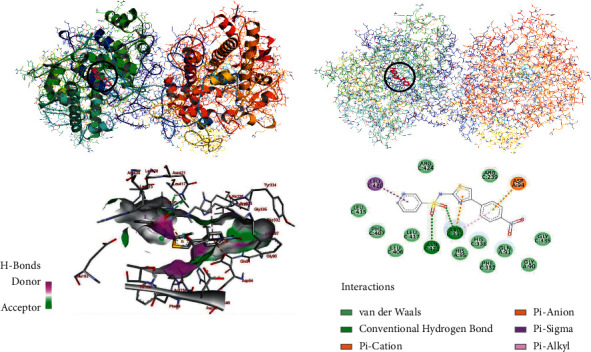
The 2D and 3D binding interactions of compound **11b** against human myeloperoxidase (PDB ID: 1DNU). 3D ribbon and line models show the binding pocket structure of human myeloperoxidase with compound **11b**. Hydrogen bonds between compounds and amino acids are shown as green dashed lines, and hydrophobic interactions are shown as pink/purple lines. Electrostatic interactions are shown as orange lines.

The molecular docking of the synthesized compounds (**7, 11a-b**) within the binding sites of human myeloperoxidase was analyzed, and the results were compared with standard antioxidant agent ascorbic acid and sulfathiazole (see Supplementary Data). The synthesized compounds (**7, 11a-b**) were found to have minimum binding energy ranging from −7.5 to –9.7 kcal/mol (Table 4). Comparing with ascorbic acid (– **8.1** kcal/mol) and sulfathiazole (– **6.9** kcal/mol), the synthesized compounds (**7, 11a-b**) have shown comparable and even better binding affinity and similar residual and DNA interaction profile with various amino acid residues. The *in silico* interaction results showed that all the synthesized compounds (**7, 11a-b**) have comparable binding affinity with ascorbic acid; among them, compounds **11a** (−9.7 kcal/mol) and **11b** (−9.6 kcal/mol) revealed good binding affinity (Figures 5 and 6). Based on the molecular docking analysis results, all the synthesized compounds have shown comparable residual interactions and comparable docking scores with ascorbic acid. Hence, these compounds might prove to be good antioxidant agents. The binding affinity, H-bond, and residual interaction of all the synthesized compounds are summarized in Table 4. The *in silico* results are in promising agreement with *in vitro* results.

### 3.5. *In Silico* Pharmacokinetics (Drug Likeness) and Toxicity Analysis

The drug likeness of the synthesized compounds (**7, 11a-b**) was characterized according to “Lipinski's rule of five.” As per Lipinski's rule, the potential molecules should have the following physicochemical properties [28], such as (i) less than 5 hydrogen bond donors (HBDs), (ii) less than 10 hydrogen bond acceptors (HBAs), (iii) a molecular mass less than 500 Da, and (iv) log P not greater than 5 and (v) total polar surface area (TPSA) should not be > 140 Å. The SwissADME computed results showed that all the synthesized compounds (**7, 11a-b**) in the present study are satisfying Lipinski's rule of five with zero violations (Table 5) [29]. Hence, all the synthesized compounds might be candidates for antioxidant and antibacterial studies. The *in silico* computed results of absorption, distribution, metabolism, and excretion (ADME) for synthesized compounds **7** and **11a-b** reference drugs sulfathiazole and ascorbic acid are given in Tables 5 and 6.

As per toxicity class classification [15, 16], none of the ligands has shown acute toxicity, and they were found similar to standard drugs. The synthesized compound **7** has shown toxicity class classification **3**, while compounds **11a** and **11b** showed much better toxicity class **5**. The toxicological prediction gives results of endpoints, such as hepatotoxicity, carcinogenicity, mutagenicity, immunogenicity, and cytotoxicity. All the synthesized compounds were predicted to be nonimmunotoxic, nonirritant, and noncytotoxic. However, compound **7** has shown mutagenicity. Pro-Tox II and OSIRIS property explorer prediction analyses have shown in Table 7. Based on ADMET prediction analysis, none of the compounds have shown acute toxicity and so might be proven as good drug candidates.

### 3.6. DFT (Density Functional Theory) Study

#### 3.6.1. Molecular Geometry

**Figure 7:**
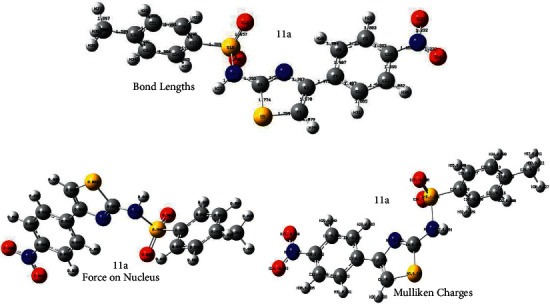
Optimized structures of compound **11a** showing bond lengths, force on nucleus, and Mulliken charges.

**Figure 8:**
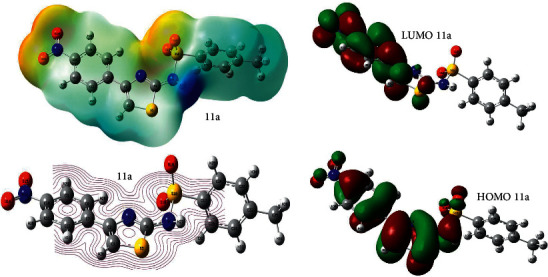
Optimized structures of compound **11a** showing electrostatic potential surface, 2D contour, and HOMO-LUMO structures.

The optimized structures of the synthesized compounds (**7, 11a-b**) along with force on nucleus, that is, 0.000, are shown in Supplementary Data. The global minimum energy obtained by the DFT structure optimization procedure for the investigated compounds is summarized in Table 8. The bond lengths, Mulliken charges, molecular electrostatic potential surface, and 2D contour, HOMO-LUMO structures for all the ligands are shown in Supplementary Data. However, parameters for the best performing ligand **11a** are shown in Figures 7 and 8.

#### 3.6.2. Frontier Molecular Orbital Analysis

The energy difference between highest occupied molecular orbital (HOMO) and lowest unoccupied molecular orbital (LUMO) is a parameter which provides excitation energy of a molecule, and it is an excellent indicator of electronic transition absorption in the molecular systems. These molecular orbitals provide insight into the reactivity nature and the physical and structural properties of molecules. The positive and negative phase is represented in red and green color in the figures. The HOMO-LUMO energies and the energy gap for the investigated compounds are calculated using B3LYP/6-31G (d,p) method. Owing to the HOMO-LUMO orbital interaction, LP-LP, and LP-bond pair type interactions were observed to be predominant in the investigated compounds according to the molecular orbital theory. The calculated HOMO-LUMO energies, the energy gap, and dipole moment are shown in Table 8.

The molecular orbital analysis for the investigated compounds based on their optimized geometry indicates that the Frontier molecular orbitals are mainly composed of *p* type-atomic orbitals. An electronic system with larger HOMO-LUMO gap should be less reactive than one with a smaller gap. Moreover, the HOMO-LUMO energy gap clearly explains the eventual charge transfer taking place within the molecule. The power of an electronegative atom in a compound to attract an electron towards was introduced by Pauling. The parameters such as hardness (*ɳ*), ionization potential (I), electronegativity (*χ*), chemical potential (*μ*), electron affinity (A), global softness (*σ*), and global electrophilicity (*ω*) are calculated.

The ionization energy (IE) can be expressed through HOMO orbital energies, and electron affinity (EA) can be expressed through LUMO orbital energies. The hardness (ɳ) corresponds to the gap between HOMO and LUMO orbital energies. The hardness has been associated with the stability of the chemical system. All the calculated values of quantum chemical parameters of the investigated molecules using the B3LYP method with 6-31G (d,p) basis set are summarized in Table 8. From the results in Table 8, it is clear that for the molecules investigated, **7** has the minimum energy gap of 3.520068 eV and **11b** has the maximum energy gap of 3.865546 eV. These facts further indicate that **7** would be more reactive among all the synthesized compounds.

#### 3.6.3. Mulliken Population Analysis

The Mulliken population analysis of the title compounds was performed at DFT-B3LYP/6-31G (d,p) level to obtain the values of the atomic charges, and the results are shown in Supplementary Data. All calculated values indicate the extensive charge delocalization in the investigated molecules. The positive charges are localized over the hydrogen atoms.

#### 3.6.4. Electric Charge Distribution and Electron Density

It is basic chemistry principle that electrons and nuclei attract each other, while electrons repel themselves and the same is the case with nuclei. In the equilibrium geometry of a molecule, these electrostatic forces just balance. The fundamentally important Hellman–Feynman theorem [30] states that the force on a nucleus in a molecule is the sum of the Coulombic forces exerted by the other nuclei and by the electron density distribution *ρ*. On the basis of these mathematical algorithms and calculations, ESP surface and 2D contours are generated through gaussian software. Molecular electrostatic potential surface and 2D contour diagrams representing electronic charge distribution are shown in Supplementary Data. It is clearly indicated that all these compounds (**7, 11a-b**) have shown balanced charge distribution, which make them adhesive towards various biological enzymes.

## 4. Conclusion

Sulfathiazole derivatives were successfully synthesized with 72–81% yield through nucleophilic substitution reaction. The synthesized compounds were fully characterized using melting point and spectroscopic techniques (^1^H and ^13^C NMR). The *in vitro* antibacterial activities of synthesized compounds were evaluated against four bacterial strains *E. coli, P. aeruginosa, S. pyogenes,* and *S. aureus* with the best activity displayed by compound **11a** against *E. coli* with an inhibition zone of 11.6 ± 0.283 and 11.1 ± 0.141 at 50 and 25 *m*g/mL, respectively. Antioxidant activity of synthesized compounds was examined. Out of the synthesized compounds, **11a** showed better % radical scavenging activity. The synthesized compounds were evaluated for their *in silico* molecular docking analysis using *S. aureus* gyrase and human myeloperoxidase. The *in silico* molecular docking analysis has shown minimum binding energy ranging from –7.8 to –10.0 kcal/mol and –7.5 to –9.7 kcal/mol using *S. aureus* gyrase and human myeloperoxidase, respectively. Compound **11a** showed very good binding score –9.7 kcal/mol with both of the proteins and had perfect alignment with *in vitro* results. Compound **11b** also showed promising binding scores with both proteins. The results of *in silico* molecular docking study of the synthesized compounds have shown comparable residual interactions and better docking scores than sulfathiazole, and all the docking results are in good agreement with *in vitro* analysis. The drug likeness of the synthesized compound satisfies Lipinski's rule of five with zero violations. Hence, all the synthesized compounds might be candidates for further *in vivo* antibacterial and antioxidant studies.

## Figures and Tables

**Table 1:** Zone of bacterial growth inhibition diameter (mm).

Compounds	Conc.	Inhibition diameter (mm)±SD			
		*E. coli*	*P*. *aeruginosa*	*S. pyogenes*	*S*. *aureus*
**7**	**50 mg/mL**	**6.95** **±** **0.777**	**7.20** **±** **1.555**	**7.35** **±** **0.636**	**6.70** **±** **0.013**
	**25 mg/mL**	**8.10** **±** **0.848**	**6.75** **±** **1.060**	**6.60** **±** **0.424**	**6.20** **±** **0.282**
	**12.5 mg/mL**	**6.35** **±** **0.495**	**6.20** **±** **0.282**	**6.00** **±** **0.011**	**6.00** **±** **0.031**

**11a**	**50 mg/mL**	**11.6** **±** **0.283**	**7.75** **±** **0.353**	**7.25** **±** **0.353**	**7.30** **±** **0.283**
	**25 mg/mL**	**11.1** **±** **0.141**	**8.15** **±** **0.495**	**10.95** **±** **0.07**	**9.15** **±** **0.495**
	**12.5 mg/mL**	**8.35** **±** **0.212**	**7.35** **±** **1.202**	**6.35** **±** **0.212**	**6.65** **±** **0.071**

**11b**	**50 mg/mL**	**7.10** **±** **0.141**	**7.80** **±** **0.015**	**6.15** **±** **0.070**	**6.45** **±** **0.495**
	**25 mg/mL**	**7.00** **±** **0.707**	**7.35** **±** **0.777**	**6.05** **±** **0.070**	**6.15** **±** **0.212**
	**12.5 mg/mL**	**7.65** **±** **0.212**	**7.50** **±** **0.141**	**6.20** **±** **0.013**	**6.65** **±** **0.212**

**Sulfamethoxazole**	**23.75 *μ*g/mL**	**15.7** **±** **0.707**	**16.7** **±** **0.636**	**15.3** **±** **2.687**	**12.8** **±** **3.252**

**Table 2:** % radical scavenging activities of synthesized compounds and ascorbic acid.

Conc. (*μ*g/ml)	Compound code							
	7		11a		11b		Ascorbic acid	
	A	% S.A	A	% S.A	A	% S.A	A	% S.A
**1. 25**	**0.13**	**71.36**	**0.056**	**87.66**	**0.133**	**70.70**	**0.025**	**94.49**
**2.5**	**0.12**	**73.56**	**0.054**	**88.10**	**0.099**	**78.19**	**0.015**	**96.69**
**5**	**0.10**	**77.97**	**0.051**	**88.76**	**0.097**	**78.63**	**0.012**	**97.35**
**10**	**0.08**	**82.37**	**0.027**	**94.05**	**0.096**	**78.85**	**0.011**	**97.57**
**IC50**		**1.918**		**1.655**		**1.927**		**1.526**

**Table 3:** Molecular docking results of synthesized compounds against *S. aureus* gyrase (PDB ID: 2XCT).

S. no.	Ligands	Binding affinity (kcal/mol)	H-bond	Residual interactions	
				Hydrophobic, electrostatic, and others	Van der Waals
**7**	**C9H7N3O2S**	**–7.8**	DT-8, DG-9, DC-12	Pi-sulfur-DT-8 Pi-sulfur-DG-9 Hydrophobic-Pi-Pi-stacked-DG-9(Dist. 3.91227) Hydrophobic-Pi-Pi-stacked-DG-9(Dist. 4.95091) Hydrophobic-Pi-Pi-stacked-DC-12 Hydrophobic-Pi-Pi-stacked-DT-8 Hydrophobic-Pi-Pi-stacked-DG-9(Dist. 3.75883) Hydrophobic-Pi-Pi-stacked-DA-13 hydrophobic-Pi-alkyl-arg-458	–

**11a**	**C16H13N3O4S2**	**–9.7**	Gly-459, DT-8, DA-13, DT-10, DA-11, Arg458 (dist. 2.76628), Arg-458 (dist. 2.8839)	Electrostatic-Pi-cation-arg-458 Hydrophobic-Pi-Pi T-shaped-DA-13 Hydrophobic-alkyl-Pro-456 Hydrophobic-Pi-alkyl-arg-458(Dist. 4.84544) Hydrophobic-Pi-alkyl-arg-458(Dist. 4.13701) hydrophobic-Pi -alkyl-Lys-417	Gly-440, Gly-441, Asp-437, DG-9

**11b**	**C15H11N3O4S2**	**–10.0**	DT-8, DA-13, DG-9 (dist. 2.61733), DG-9 (Dist. 3.15352), DG-9 (Dist. 3.09909)	Pi-sulfur-DA-13 Hydrophobic-Pi-Pi-stacked-DG-9(Dist. 5.76015) Hydrophobic-Pi-Pi-stacked-DG-9(Dist. 4.6504) Hydrophobic-Pi-Pi-stacked-DT-8 Hydrophobic-Pi-Pi T-shaped-DA-13 Hydrophobic-Pi-alkyl-arg-458(Dist. 4.46267) Hydrophobic-Pi-alkyl-arg-458(Dist. 4.38817)	Arg-1122, Gly-459, Asp-437, DT-10, DA11
	**Sulfathiazole (C9H9N3O2S2)**	**–7.4**	Arg-1122, Asp-508, DT-8	Electrostatic-attractive charge-arg-1122 Pi-sulfur-Phe-1123 Hydrophobic-Pi-Pi-stacked-DG-9(Dist. 4.26823) Hydrophobic-Pi-Pi-stacked-DG-9(Dist. 5.42495) Hydrophobic-Pi-Pi-stacked-DT-8 Hydrophobic-Pi-Pi T-shaped-Phe-1123	Mn-2492, Glu-435, Gly-436, Gly-1082, Ser-1084

DA = deoxyadenosine; DG = deoxyguanosine; DT = deoxythymidine; DC = deoxycytidine.

**Table 4:** Molecular docking results of synthesized compounds against Human myeloperoxidase (PDB ID: 1DNU).

S. no.	Ligands	Binding affinity (kcal/mol)	H-bond	Residual interactions	
				Hydrophobic, electrostatic, and others	Van der Waals
**7**	**C9H7N3O2S**	**−7.5**	Arg-239, Arg-333, Phe-332	Electrostatic-Pi-anion-asp-94 Hydrophobic-amide-Pi stacked-gly-90:C, O; Gln-91: N Hydrophobic-Pi-alkyl-arg-333	Gln-91, His-336, His-95, Tyr-296, Tyr-334, Gly-335

**11a**	**C16H13N3O4S2**	**−9.7**	Ala-35, Ile-160, Pro-34, Arg-31 (dist.2.62254), Arg-31 (dist. 3.5946), Arg-323 (Dist. 2.10377), Arg-323 (dist. 2.48768), Arg-323 (dist. 2.41168)	Hydrophobic-Pi-Pi T-shaped-UNK0 Hydrophobic-Pi-alkyl-Ala-28 Hydrophobic-Pi-alkyl-Ile-160 Hydrophobic-Pi-alkyl-arg-31(Dist. 4.12533) Hydrophobic-Pi-alkyl-arg-31(Dist. 4.79992)	Val-30, Ala-35, Thr-159, Asn-162
**11b**	**C15H11N3O4S2**	**−9.6**	Arg-333 (dist. 2.4154), Arg-333 (2.53959), Asn-421 (dist. 3.00117), Asn-421 (dist. 3.08562)	Electrostatic-Pi-cation-arg-333 Electrostatic-Pi-anion-asp-94 Hydrophobic-Pi-sigma-leu-420 Hydrophobic-Pi-alkyl-arg-333m	Arg-424, Arg-239, Leu-415, Phe-407, Leu-417, Leu-406, His-95, His-336, Gln-91, Phe-332, Gly-335, Gly-90
	**Sulfathiazole (C9H9N3O2S2)**	**−6.9**	Arg-333, Asp-98	Electrostatic-attractive charge-arg-333 Electrostatic-Pi-cation-arg-239 Electrostatic-Pi-cation-arg-333 Electrostatic-Pi-anion-asp-94(Dist. 4.49766) electrostatic-Pi-anion-asp-94(dist. 4.85444) Pi-sulfur-His-95 Pi-sulfur-His-336 Hydrophobic-Pi-Pi T-shaped-His-336 hydrophobic-Pi-alkyl-arg-333	Phe-99, Thr-100, Thr-329, Gln-91, Gly-335, Phe-332
	**Ascorbic acid (C**_**6**_ **H**_**8**_ **O**_**6**_ **)**	**−8.1**	Gln-91, Thr-100, Arg-239: HH21, Arg-239:CD, Arg-333: HE, Arg-333: HH11, Arg333:CA	Electrostatic-Pi-anion-asp-94 Hydrophobic-Pi-alkyl-arg-333	His-336, Phe-332, Phe-99, Thr-329, His-95, Asp-98

**Table 5:** Drug likeness predictions of compounds, computed by SwissADME.

S. no.	Ligands	Mol. Wt. (g/mol)	NHD	NHA	NRB	TPSA (A^°2^)	Log *P* (iLOGP) lipophilicity	Log *S* (ESOL) water solubility	Synthetic accessibility	Lipinski's rule of five with zero violations
**7**	**C9H7N3O2S**	221.24	1	3	2	112.97	1.13	−3.15	2.28	0
**11a**	**C16H13N3O4S2**	375.42	1	5	5	141.50	1.97	−4.65	3.21	0
**11b**	**C15H11N3O4S2**	361.40	1	5	5	141.50	2.04	−4.35	3.10	0
** **	**Sulfathiazole (C9H9N3O2S2)**	255.32	2	3	3	121.70	0.69	−1.77	2.80	0
	**Ascorbic acid (C**_**6**_ **H**_**8**_ **O**_**6**_ **)**	176.12	4	6	2	107.22	-0.31	0.23	3.47	0

NHD = number of hydrogen donors, NHA = number of hydrogen acceptors, NRB = number of rotatable bonds, and TPSA = total polar surface area.

**Table 6:** ADME predictions of compounds, computed by SwissADME and PreADMET.

S. No.	Ligands	Skin Permeation value (log *Kp*) cm/s	GI absorption	BBBPermeability	Inhibitor Interaction					
					Pgp substrate	CYP1A2 inhibitor	CYP2C19 inhibitor	CYP2C9 inhibitor	CYP2D6 inhibitor	CYP3A4 inhibitor
**7**	**C9H7N3O2S**	-5.92	High	No	No	Yes	Yes	No	No	No
**11a**	**C16H13N3O4S2**	-5.98	Low	No	No	Yes	Yes	Yes	No	Yes
**11b**	**C15H11N3O4S2**	-6.16	Low	No	No	Yes	Yes	Yes	No	Yes
** **	**Sulfathiazole (C9H9N3O2S2)**	-7.82	High	No	No	No	No	No	No	No
	**Ascorbic acid (C**_**6**_ **H**_**8**_ **O**_**6**_ **)**	-8.54	High	No	No	No	No	No	No	No

GI = gastrointestinal, BBB = blood brain barrier, P-gp = P-glycoprotein, and CYP = cytochrome-P.

**Table 7:** Toxicity prediction of compounds, computed by ProTox-II and OSIRIS property explorer.

S. no.	Ligands	LD50 (mg/kg)	Toxicity class	Organ toxicity					
				Hepatotoxicity	Carcinogenicity	Immunotoxicity	Mutagenicity	Cytotoxicity	Irritant
**7**	**C9H7N3O2S**	300	3	Active	Active	Inactive	Active	Inactive	No
**11a**	**C16H13N3O4S2**	4500	5	Active	Active	Inactive	Inactive	Inactive	No
**11b**	**C15H11N3O4S2**	4500	5	Active	Active	Inactive	Inactive	Inactive	No
** **	**Sulfathiazole (C9H9N3O2S2)**	4500	5	Active	Active	Inactive	Inactive	Inactive	No
	**Ascorbic acid (C**_**6**_ **H**_**8**_ **O**_**6**_ **)**	3367	5	Inactive	Inactive	Inactive	Inactive	Inactive	No

**Table 8:** The various quantum chemical parameters of synthesized compounds.

S. no.	Compounds	Optimized energy (Hartree)	EHOMO (eV)	ELUMO (eV)	Energy Gap ΔE (eV)	Electronegativity χ (eV)	Pauling hardness *η* (eV)	Global softness ∑ (eV^−1^)	Global electrophilicity ω (eV)	Dipole moment (Debye)
**7**	**C9H7N3O2S**	−1059.9841	−5.845345	−2.325277	3.520068	4.085311	1.760034	0.568171	4.741319	7.8488217
**11a**	**C16H13N3O4S2**	−1878.9157	−6.181719	−2.334937	3.846781	4.258328	1.923391	0.519915	4.713903	11.772033
**11b**	**C15H11N3O4S2**	−1839.5939	−6.224621	−2.359075	3.865546	4.291848	1.932773	0.517391	4.765164	11.133554

## Data Availability

The data used to support the findings of this study are included within the manuscript and also submitted as supporting information and, if needed more, can be asked to submit more by corresponding author.

## Supplementary Materials

Supplementary Materials
^1^H NMR, ^13^C NMR, DEPT-135 NMR Spectra of synthesized compounds, the 2D and 3D binding interactions of sulfathiazole and all synthesized compounds (7, 11a-b) against S. aureus gyrase (PDB ID :2XCT), the 2D and 3D binding interactions of sulfathiazole, ascorbic acid, and all synthesized compounds (7, 11a-b) against human myeloperoxidase (PDB ID: 1DNU), optimized structures of all synthesized compounds (7, 11a-b) showing force on nucleus, bond lengths, Mulliken charges, molecular electrostatic potential surface, 2D contour, HOMO-LUMO structures and optimized parameters of synthesized compounds computed through Gaussian (R) 09 program are shown in Supplementary Materials.
