# Supplementary material for: Synthesis, DFT Analysis, and Evaluation of Antibacterial and Antioxidant Activities of Sulfathiazole Derivatives Combined with In Silico Molecular Docking and ADMET Predictions
Source: Biochem Res Int. 2021 Dec 14;2021:7534561. doi: 10.1155/2021/7534561 (PMC8692053; doi:10.1155/2021/7534561)
Supplement: Supplementary Materials — 1H NMR, 13C NMR, DEPT-135 NMR Spectra of synthesized compounds, the 2D and 3D binding interactions of sulfathiazole and all synthesized compounds (7, 11a-b) against S. aureus gyrase (PDB ID :2XCT), the 2D and 3D binding interactions of sulfathiazole, ascorbic acid, and all synthesized compounds (7, 11a-b) against human myeloperoxidase (PDB ID: 1DNU), optimized structures of all synthesized compounds (7, 11a-b) showing force on nucleus, bond lengths, Mulliken charges, molecular electrostatic potential surface, 2D contour, HOMO-LUMO structures and optimized parameters of synthesized compounds computed through Gaussian (R) 09 program are shown in Supplementary Materials. [file 7534561.f1.docx]

**Synthesis, DFT Analysis, and Evaluation of Antibacterial and Antioxidant Activities of Sulfathiazole Derivatives combined with *in-silico* molecular docking and ADMET predictions**

Yoseph Samuel^1^, Ankita Garg^1^, Endale Mulugeta^1^*

^1^Department of Applied Chemistry, School of Applied Natural Science, Adama Science and Technology University, P.O.Box 1888, Adama, Ethiopia

*Email: Endale Mulugeta; [endexindex05@gmail.com](mailto:endexindex05@gmail.com)

**Table of Contents**

1. ^1^H NMR, ^13^C NMR and DEPT-135 NMR Spectra of all compounds….........................S2-S10.
2. The 2D and 3D binding interactions of Sulfathiazole and all synthesized compounds (**7, 11a-b**) against *S. aureus* gyrase (PDB ID: 2XCT).…………………………………….…..S11-S14.
3. The 2D and 3D binding interactions of Sulfathiazole, Ascorbic Acid and all synthesized compounds (**7, 11a-b**) against *human* *myeloperoxidase* (PDB ID: 1DNU)…………..S15-S19.
4. Optimized structures of all synthesized compounds (7, 11a-b) showing Force on Nucleus, Bond lengths, Mulliken Charges, Molecular electrostatic Potential Surface, 2D Contour and HOMO-LUMO Structures………………………………………………………….....S20-S25.
5. Optimized Parameters of synthesized compounds computed through Gaussian (R) 09 program……………………………………………………………….……………….S26-S33.

**NMR spectra of synthesized compounds (7, 11a-b)**

**Figure S1**: ^1^H-NMR (400 MHz, DMSO) spectrum of compound **7**

**Figure S2**: ^13^C-NMR (100 MHz, DMSO) spectrum of compound **7**

**Figure S3:** DEPT-135 (100 MHz, DMSO) spectrum of compound **7**

**Figure S4**: ^1^H-NMR (400 MHz, DMSO) spectrum of compound **11a**

**Figure S5:** ^13^C-NMR (100 MHz, DMSO) spectrum of compound **11a**

**Figure S6:** DEPT-135 (100 MHz, DMSO) spectrum of compound **11a**

**Figure S7:** ^1^H-NMR (400 MHz, DMSO) spectrum of compound **11b**

**Figure S8:** ^13^C-NMR (100 MHz, DMSO) spectrum of compound **11b**

**Figure S9:** DEPT-135 (100 MHz, DMSO) spectrum of compound **11b**


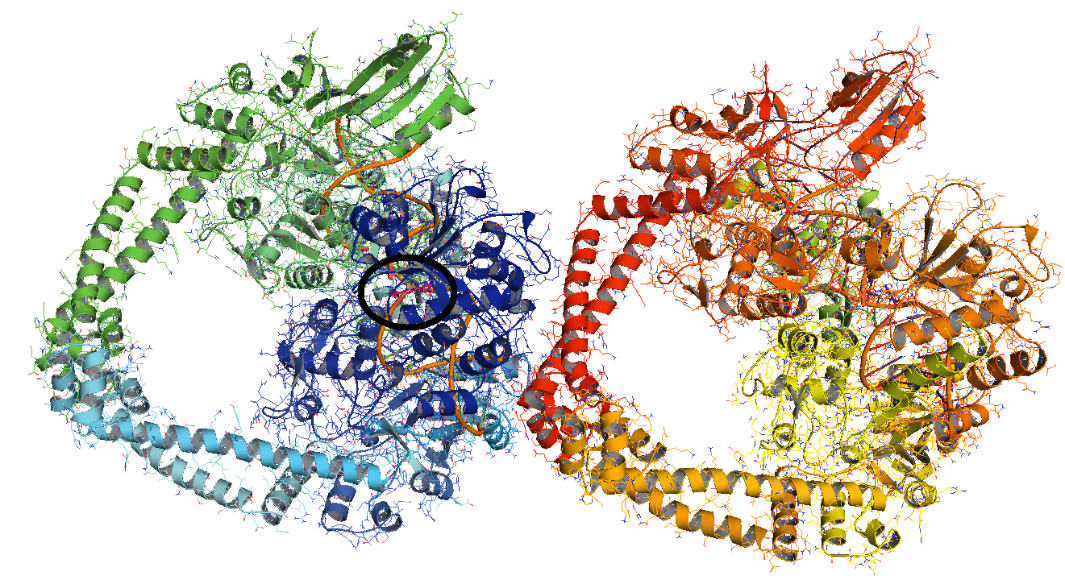

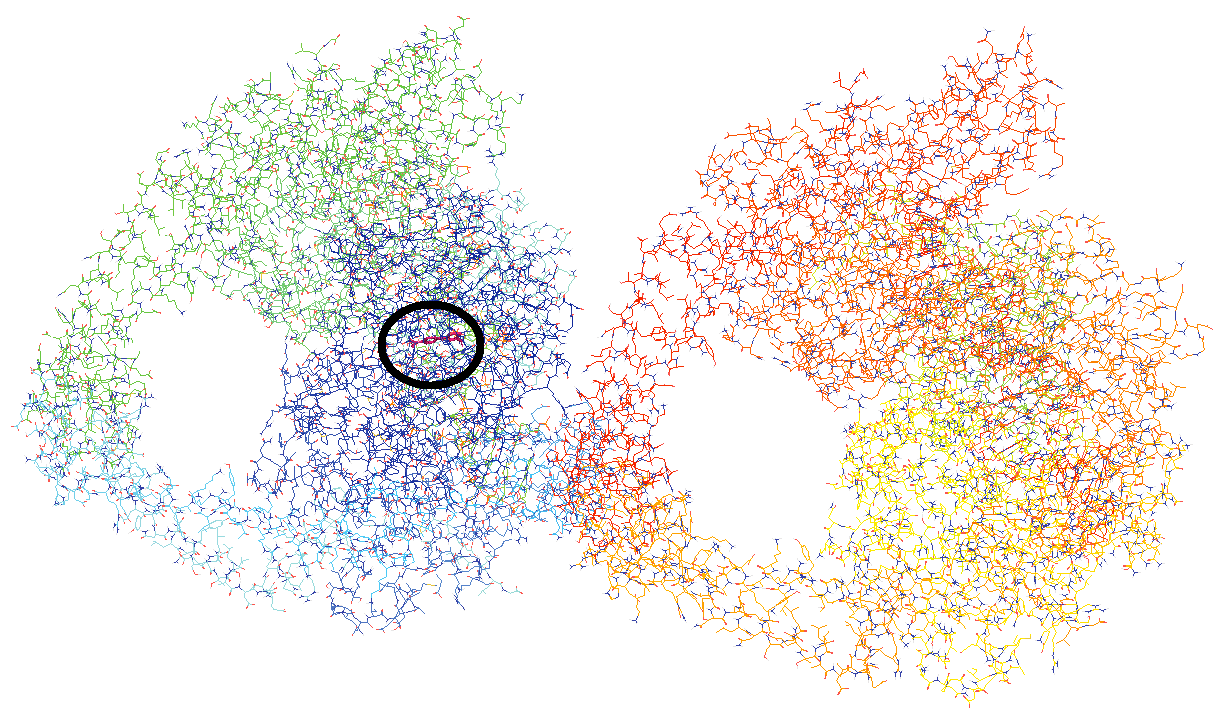


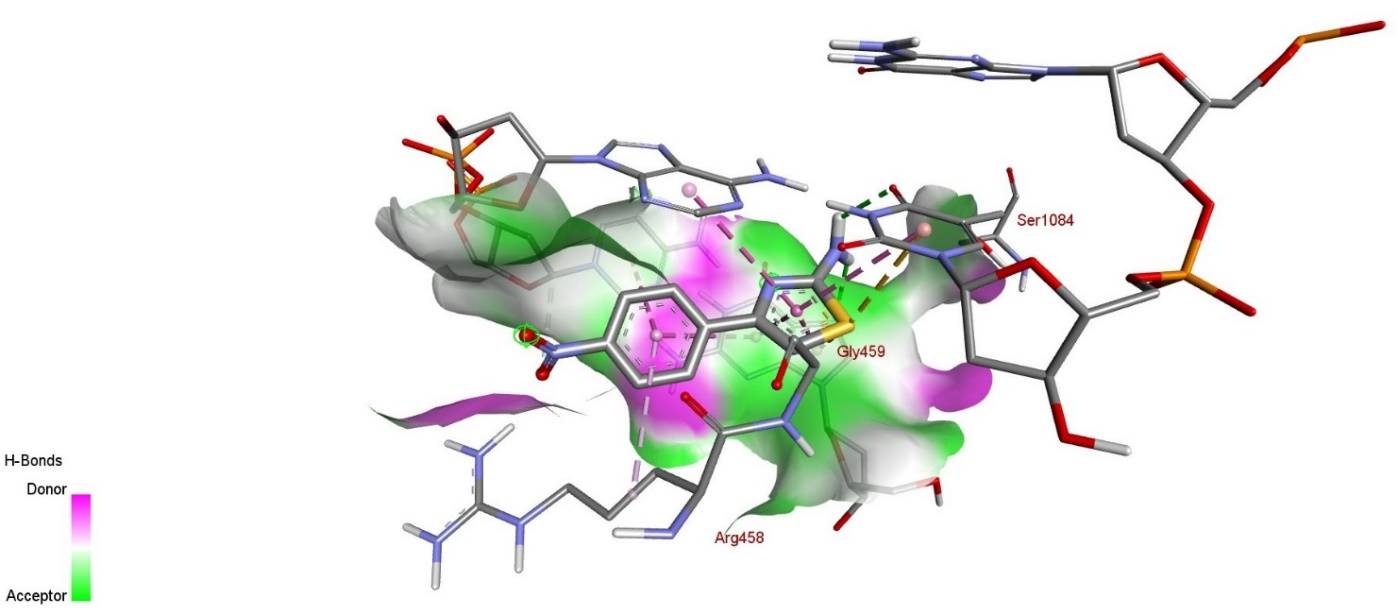


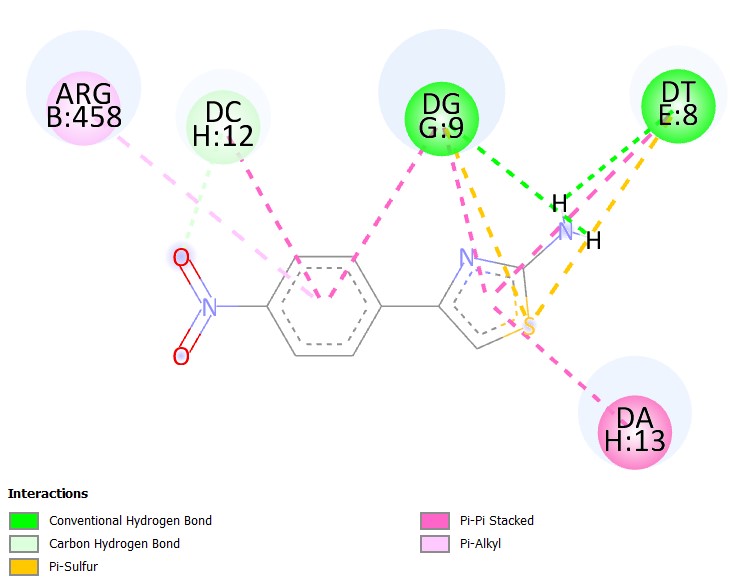


**Figure S10:** The 2D and 3D binding interactions of compound 7 against *S. aureus* Gyrase (PDB ID: 2XCT). 3D Ribbon and line models show the binding pocket structure of *S. aureus* Gyrase with compound 7. Hydrogen bond between compounds and amino acids are shown as green dash lines, hydrophobic interactions are shown as pink lines. Electrostatic interactions are shown as orange lines.


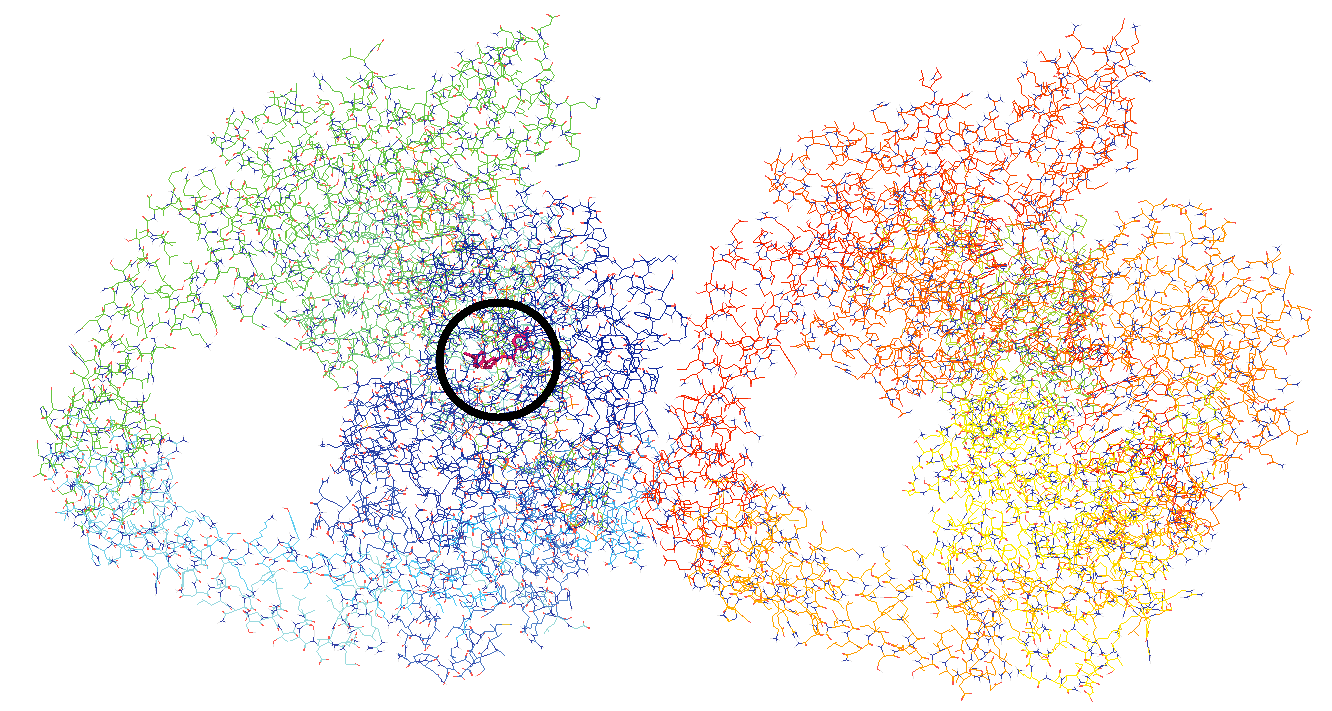

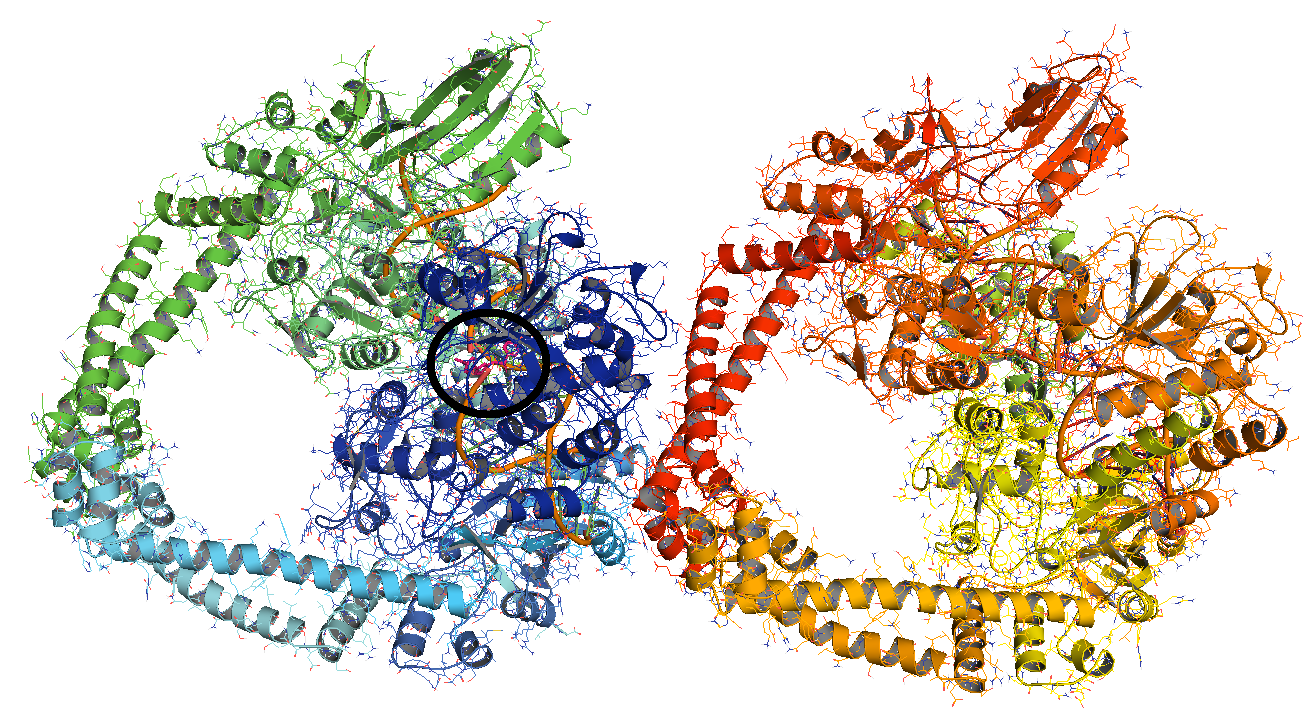


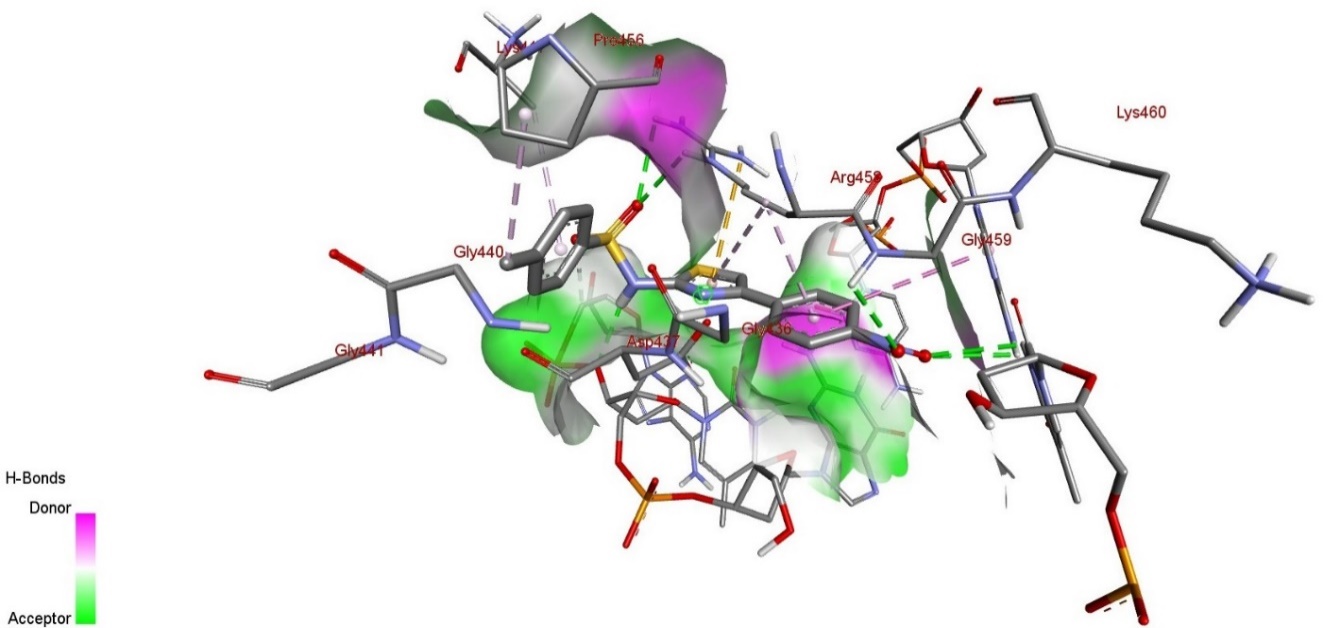


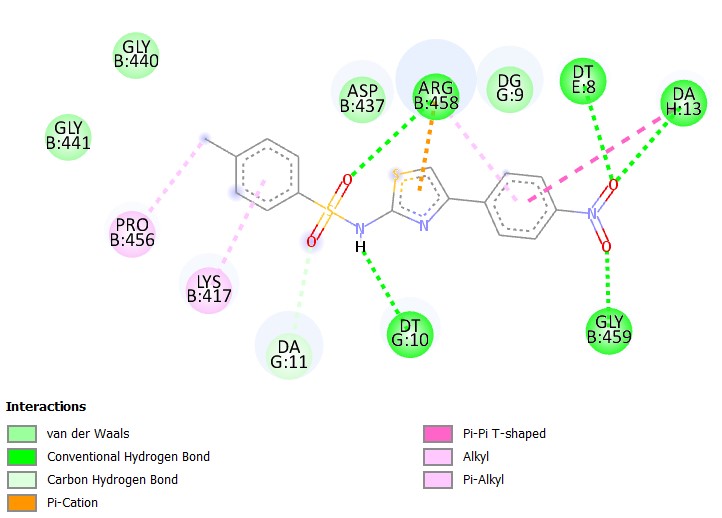


**Figure S11:** The 2D and 3D binding interactions of compound 11a against *S. aureus* Gyrase (PDB ID: 2XCT). 3D Ribbon and line models show the binding pocket structure of *S. aureus* Gyrase with compound 11a. Hydrogen bond between compounds and amino acids are shown as green dash lines, hydrophobic interactions are shown as pink lines. Electrostatic interaction is shown as orange line.


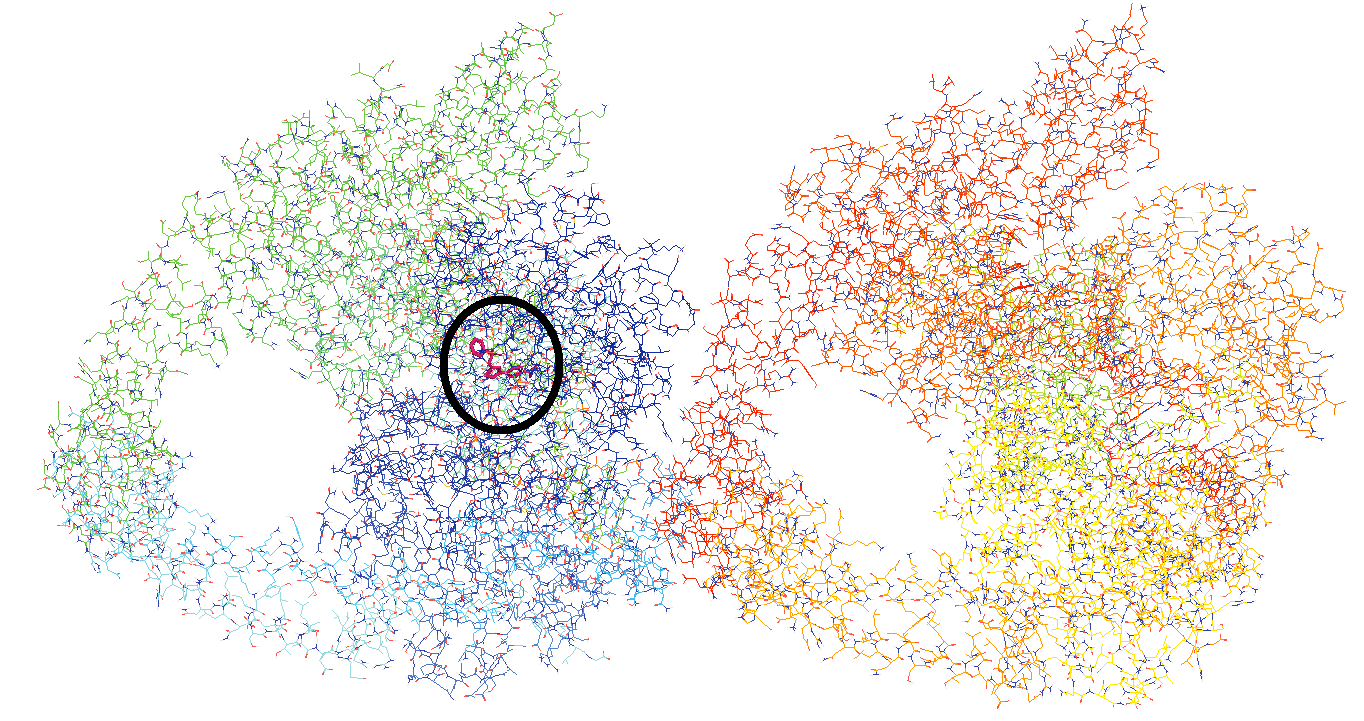

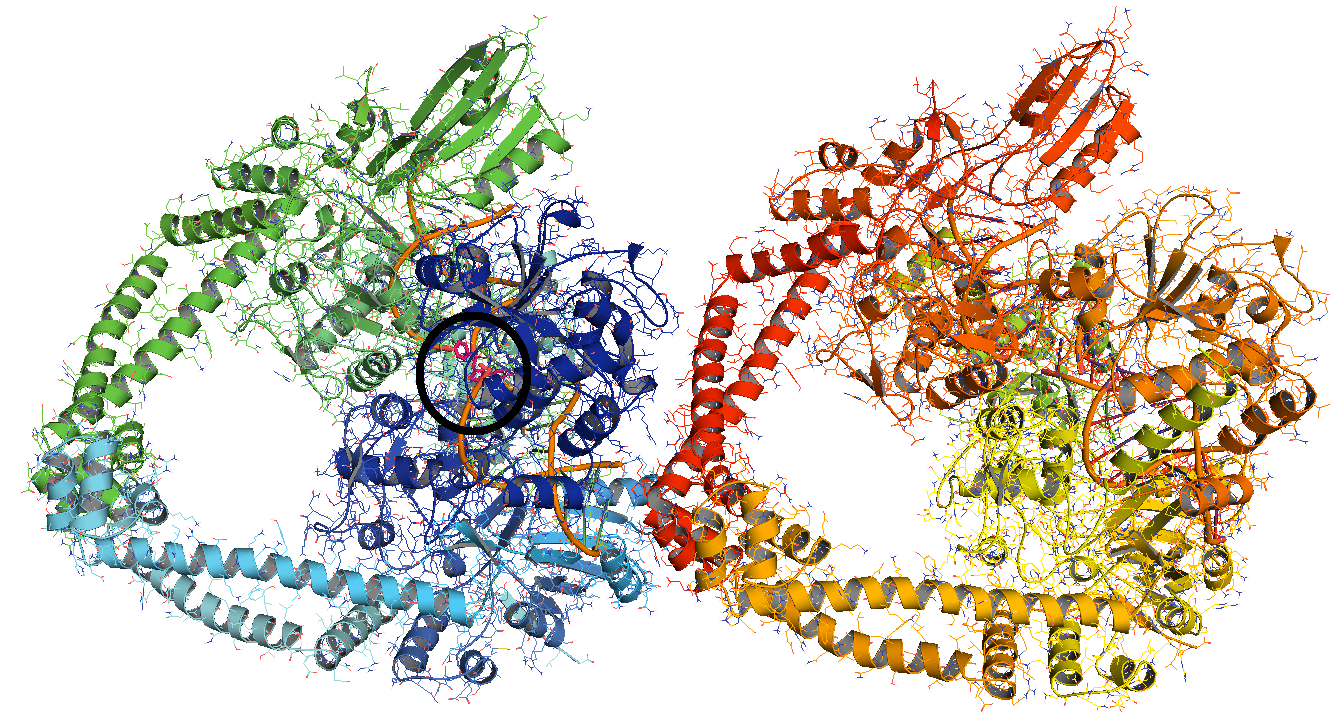


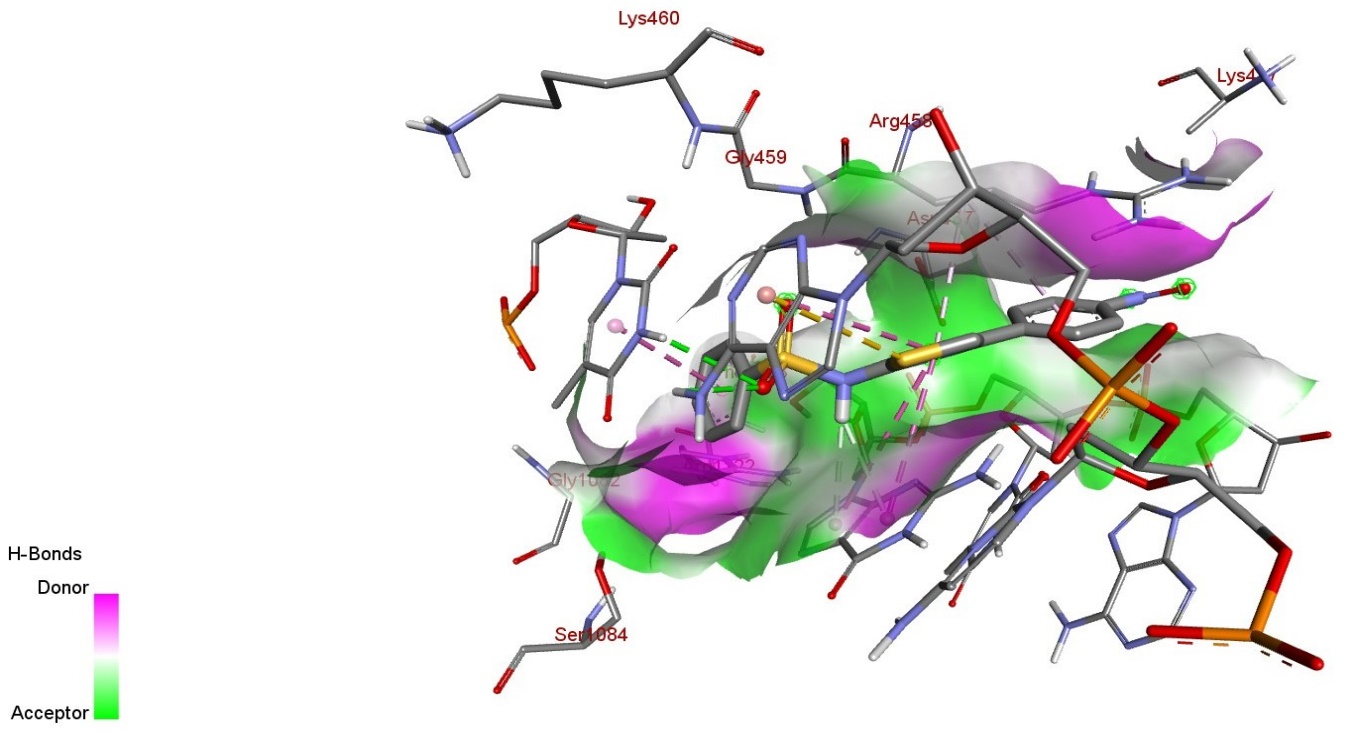


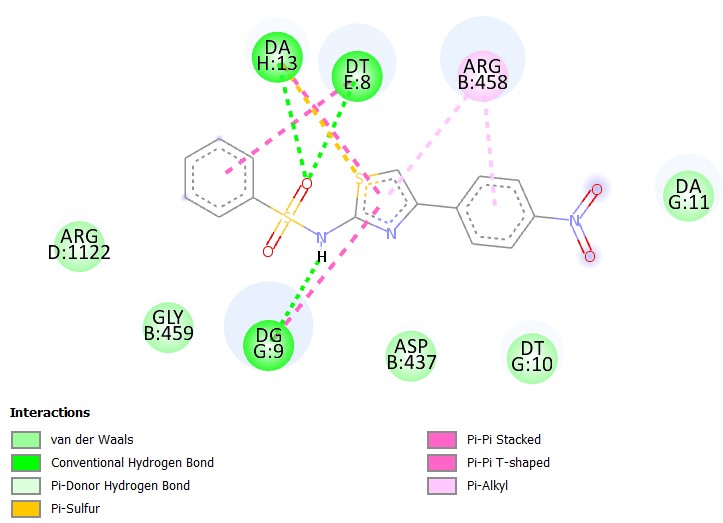


**Figure S12:** The 2D and 3D binding interactions of compound 11b against *S. aureus* Gyrase (PDB ID: 2XCT). 3D Ribbon and line models show the binding pocket structure of *S. aureus* Gyrase with compound 11b. Hydrogen bond between compounds and amino acids are shown as green dash lines, hydrophobic interactions are shown as pink/purple lines. Electrostatic interaction is shown as orange line.


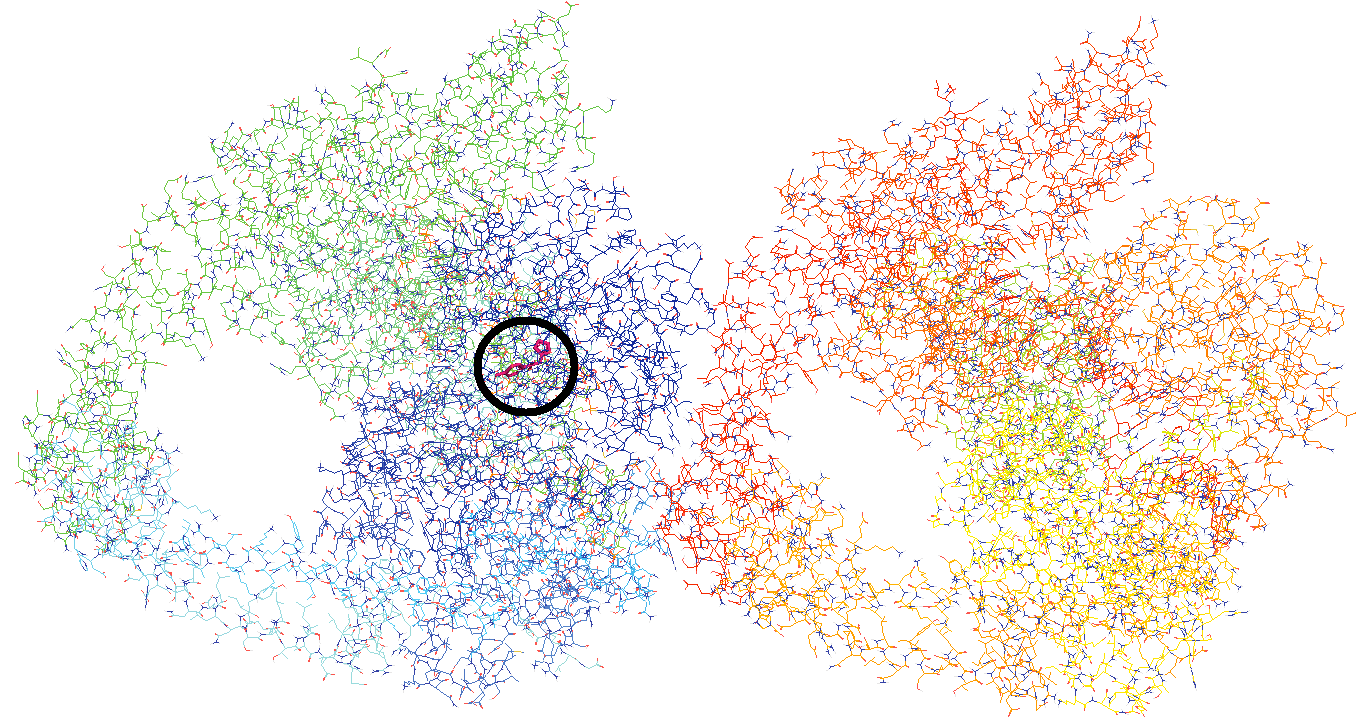

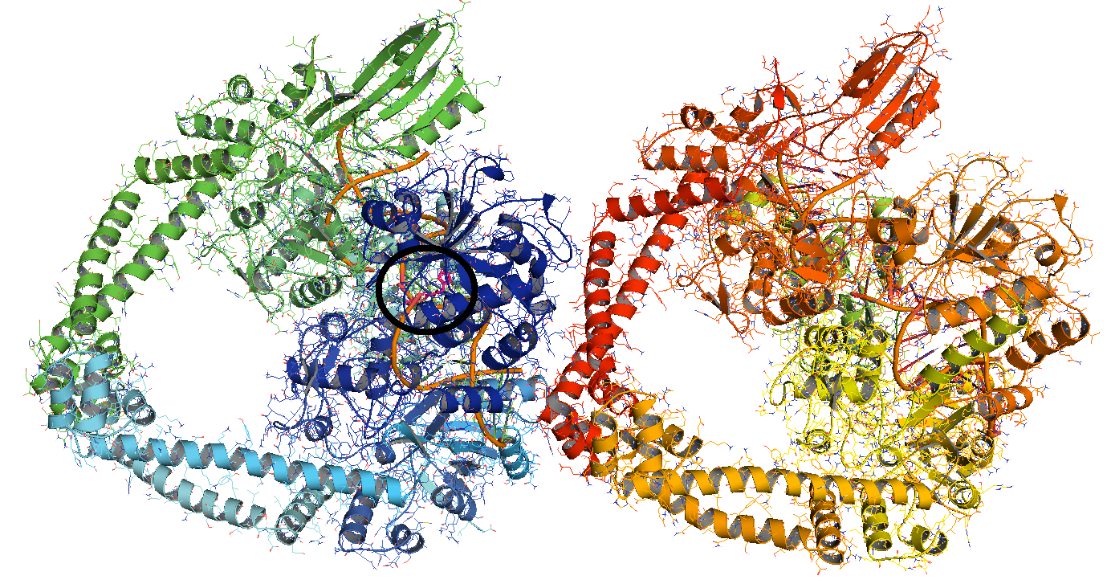


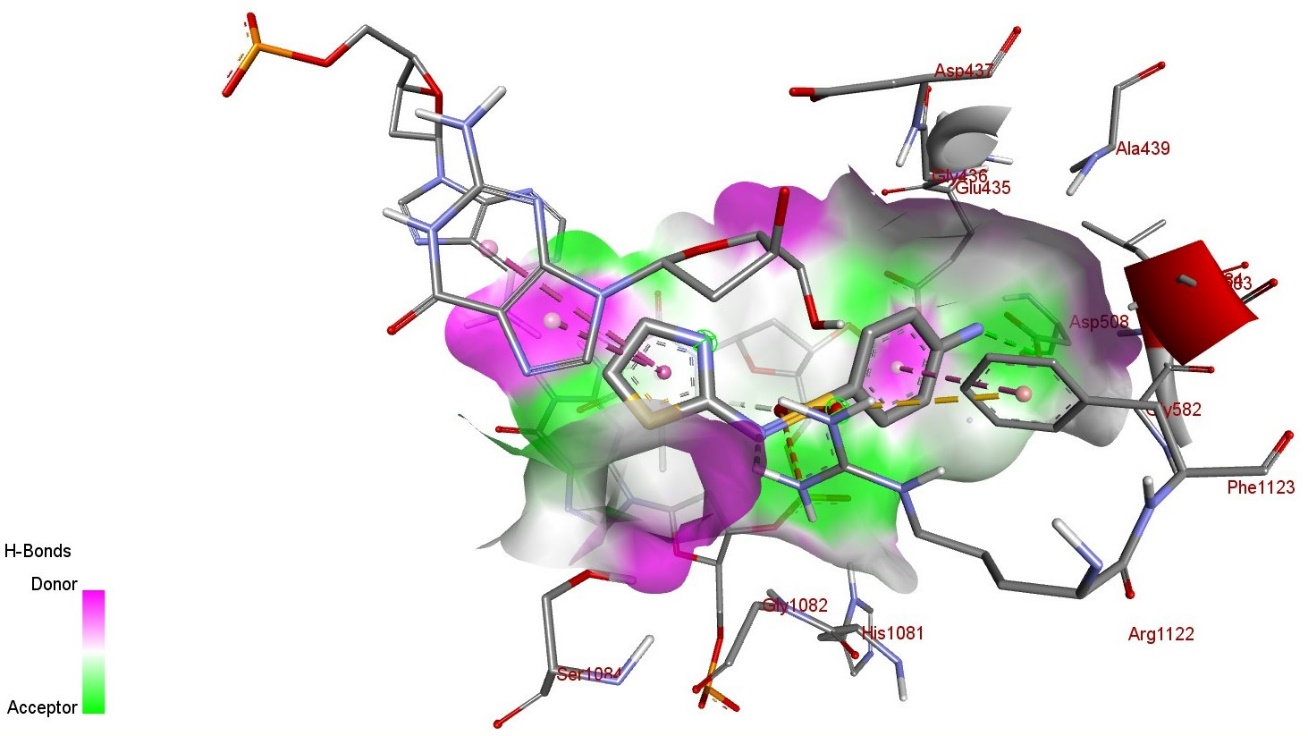


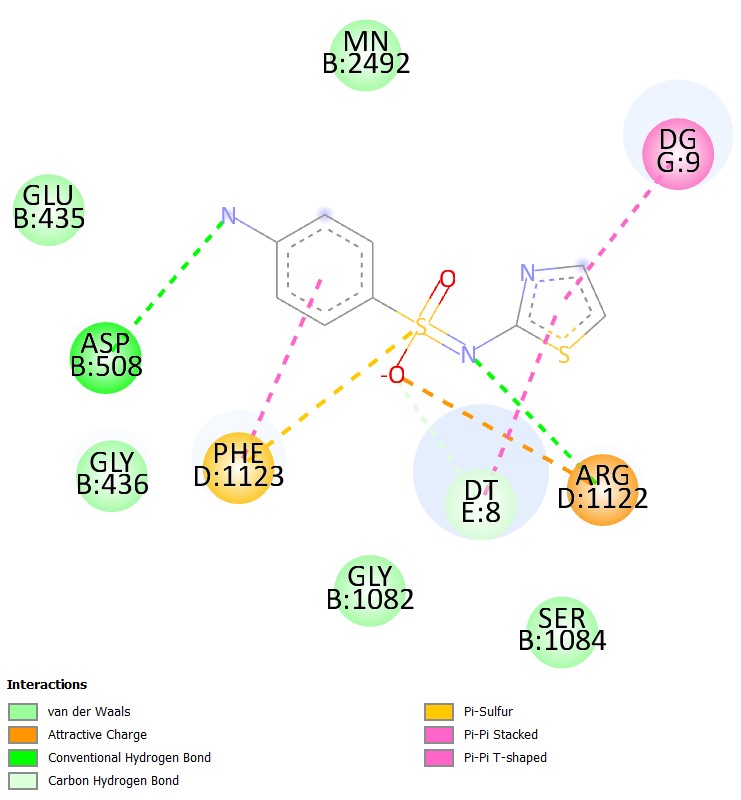


**Figure S13:** The 2D and 3D binding interactions of reference drug Sulfathiazole against *S. aureus* Gyrase (PDB ID: 2XCT). 3D Ribbon and line models show the binding pocket structure of *S. aureus* Gyrase with Sulfathiazole. Hydrogen bond between compounds and amino acids are shown as green dash lines, hydrophobic interactions are shown as pink lines. Electrostatic interactions are shown as orange lines.


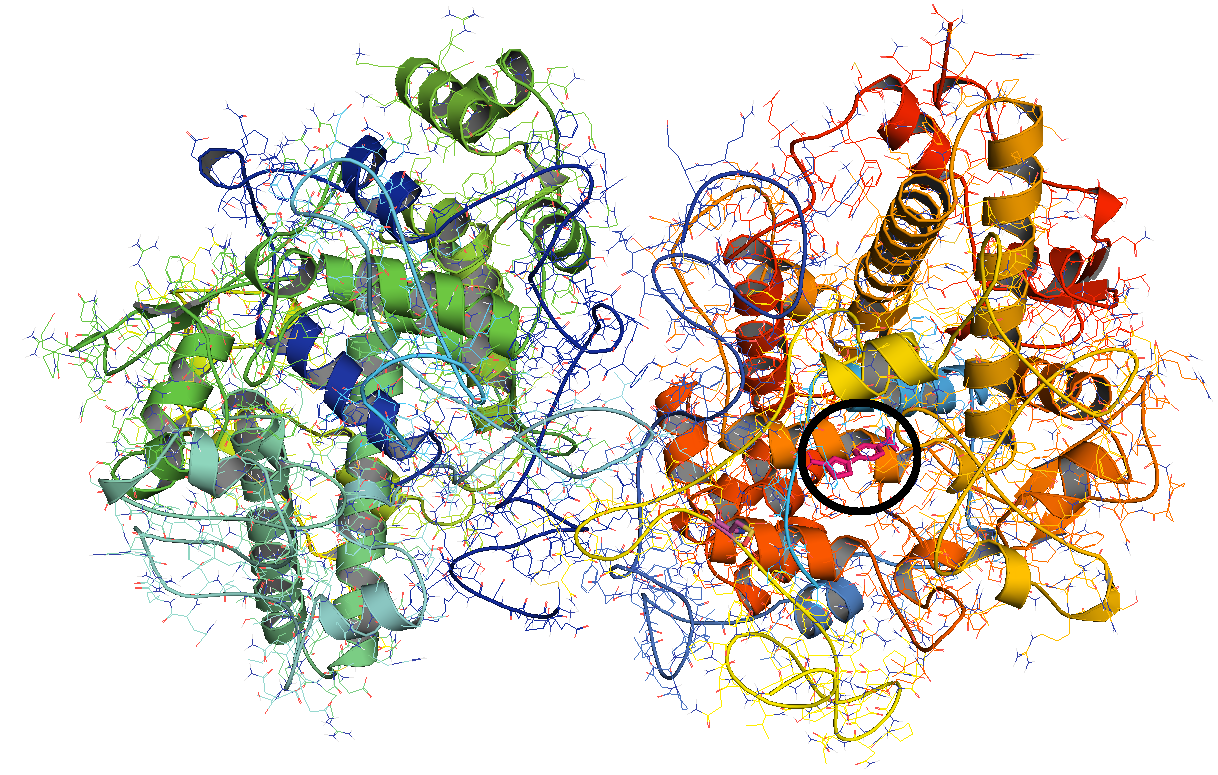

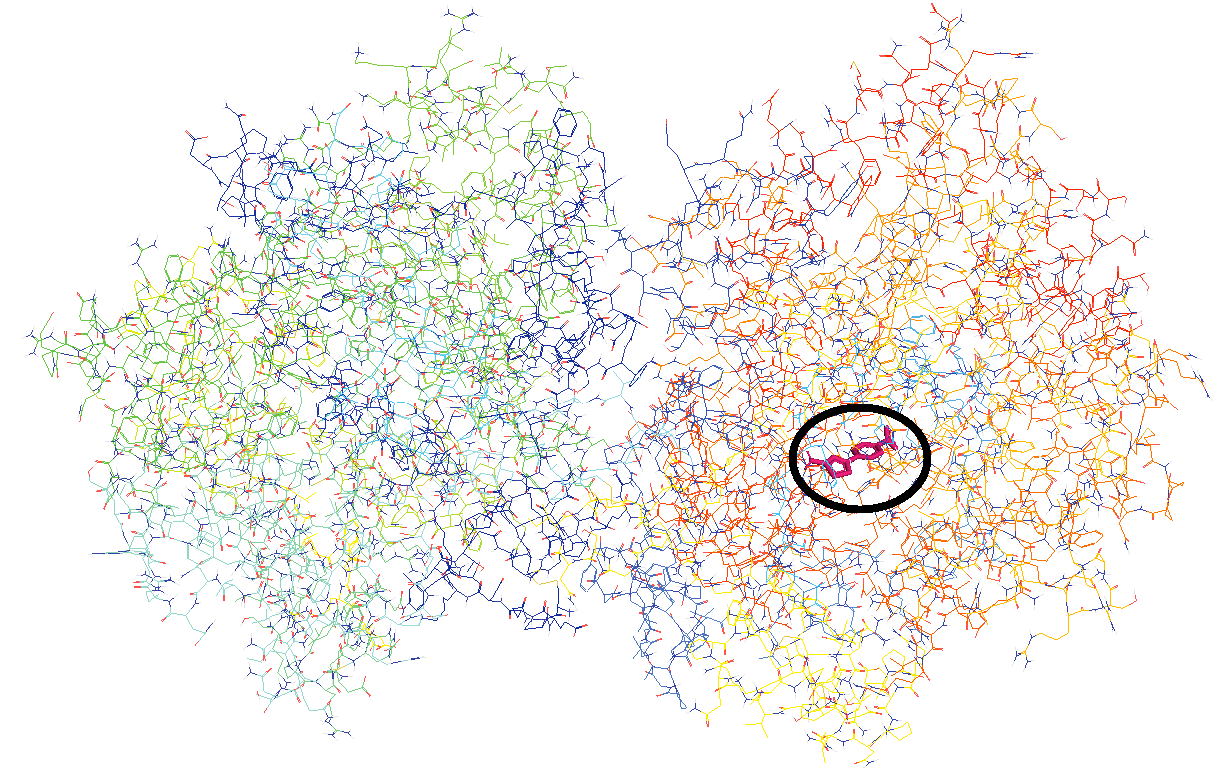


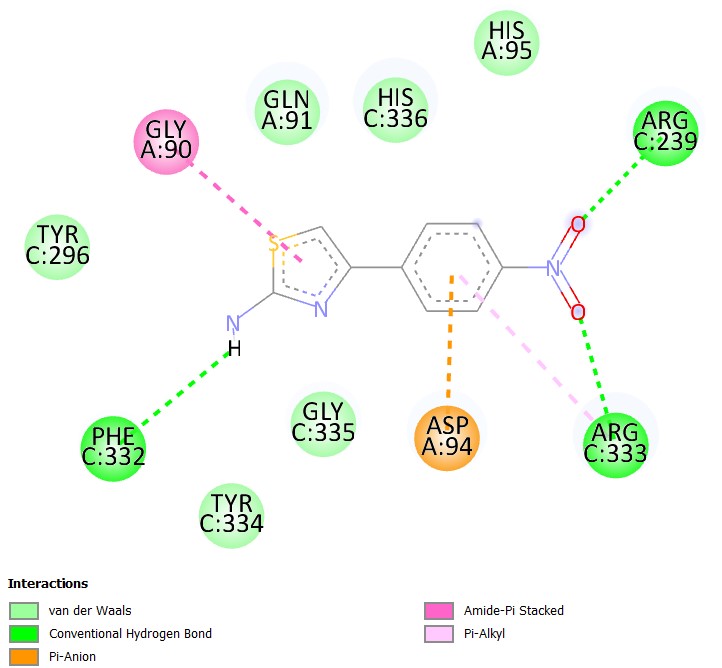


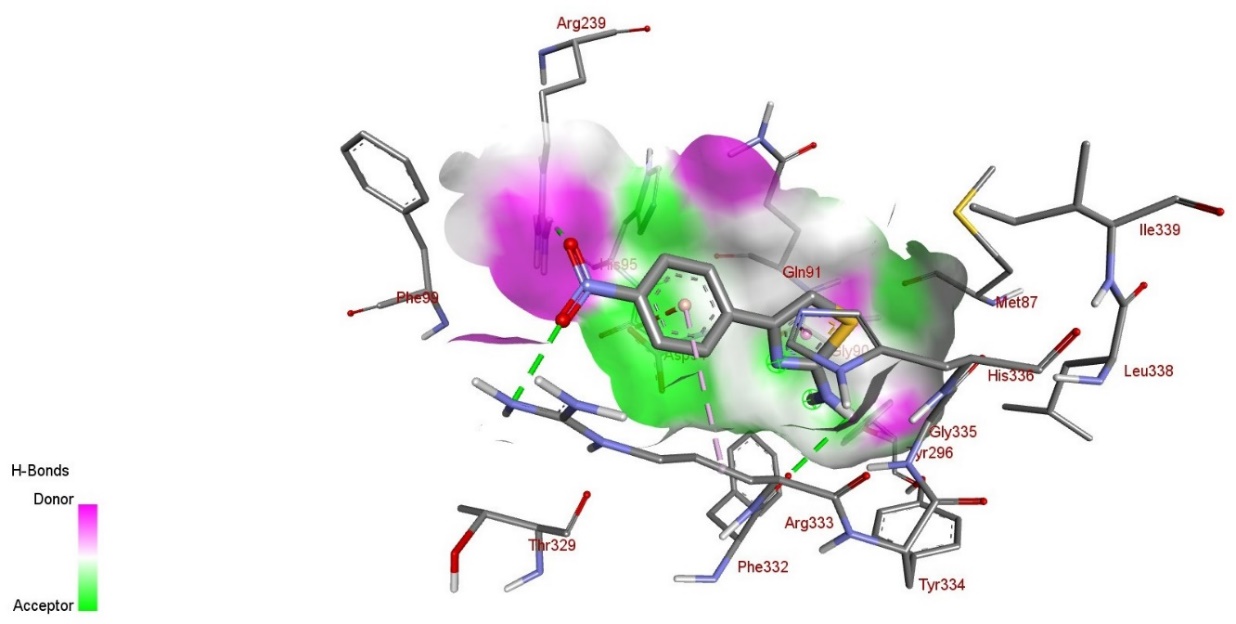


**Figure S14:** The 2D and 3D binding interactions of compound 7 against human myeloperoxidase (PDB ID: 1DNU). 3D Ribbon and line models show the binding pocket structure of human myeloperoxidase with compound 7. Hydrogen bond between compounds and amino acids are shown as green dash lines, hydrophobic interactions are shown as pink lines. Electrostatic interaction is shown as orange line.


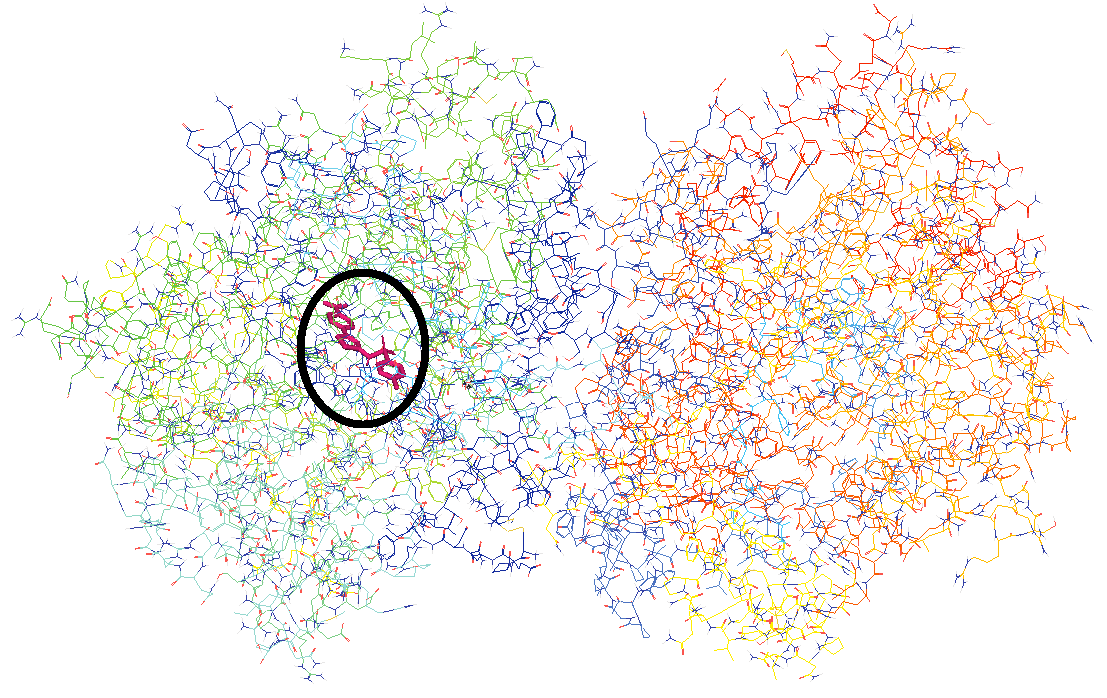

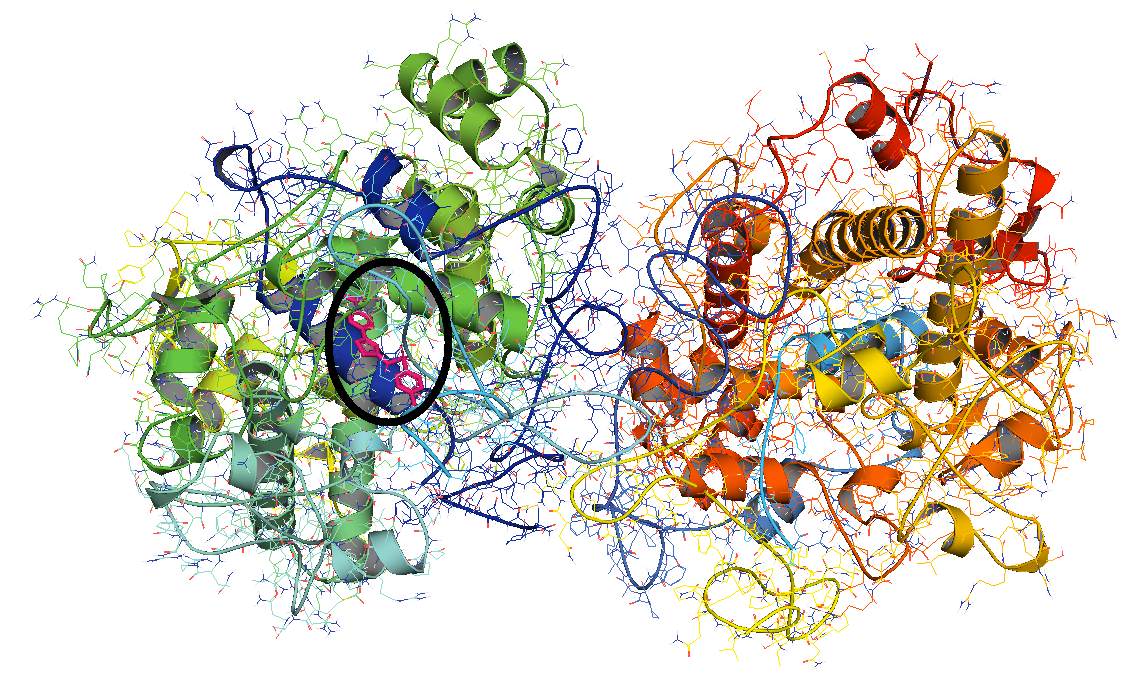


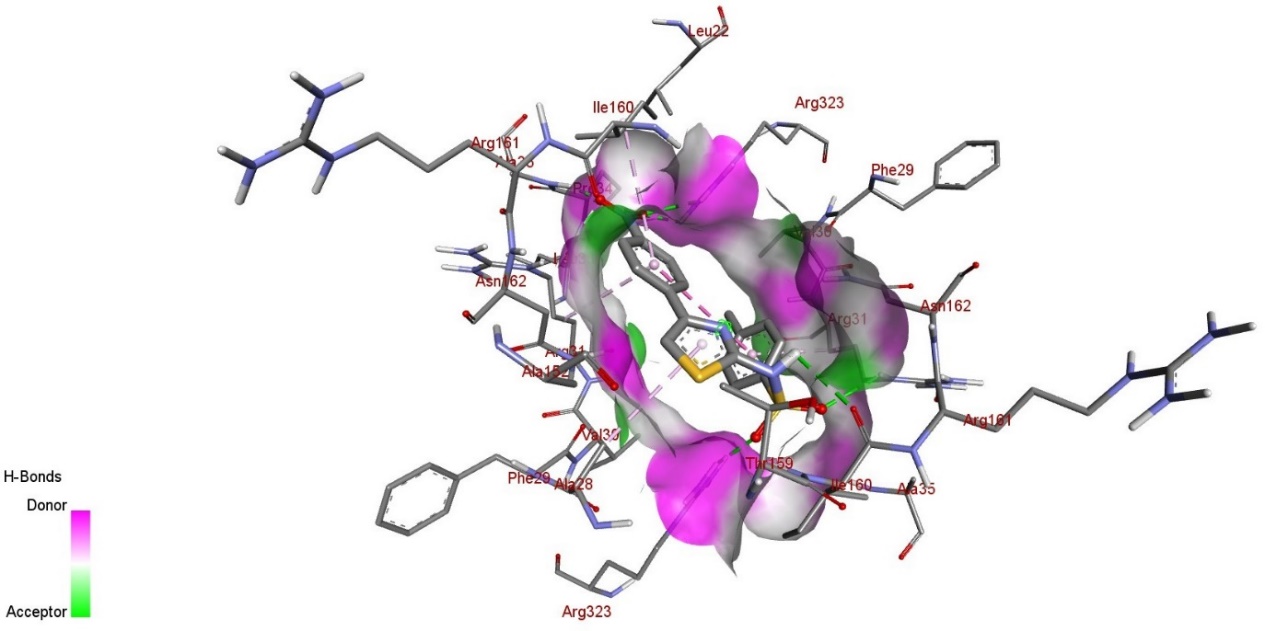


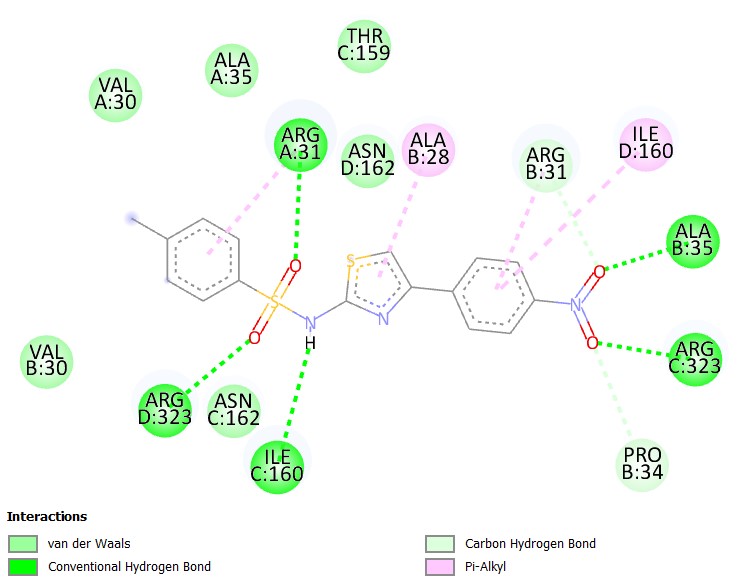


**Figure S15:** The 2D and 3D binding interactions of compound 11a against human myeloperoxidase (PDB ID: 1DNU). 3D Ribbon and line models show the binding pocket structure of human myeloperoxidase with compound 11a. Hydrogen bond between compounds and amino acids are shown as green dash lines, hydrophobic interactions are shown as pink lines.


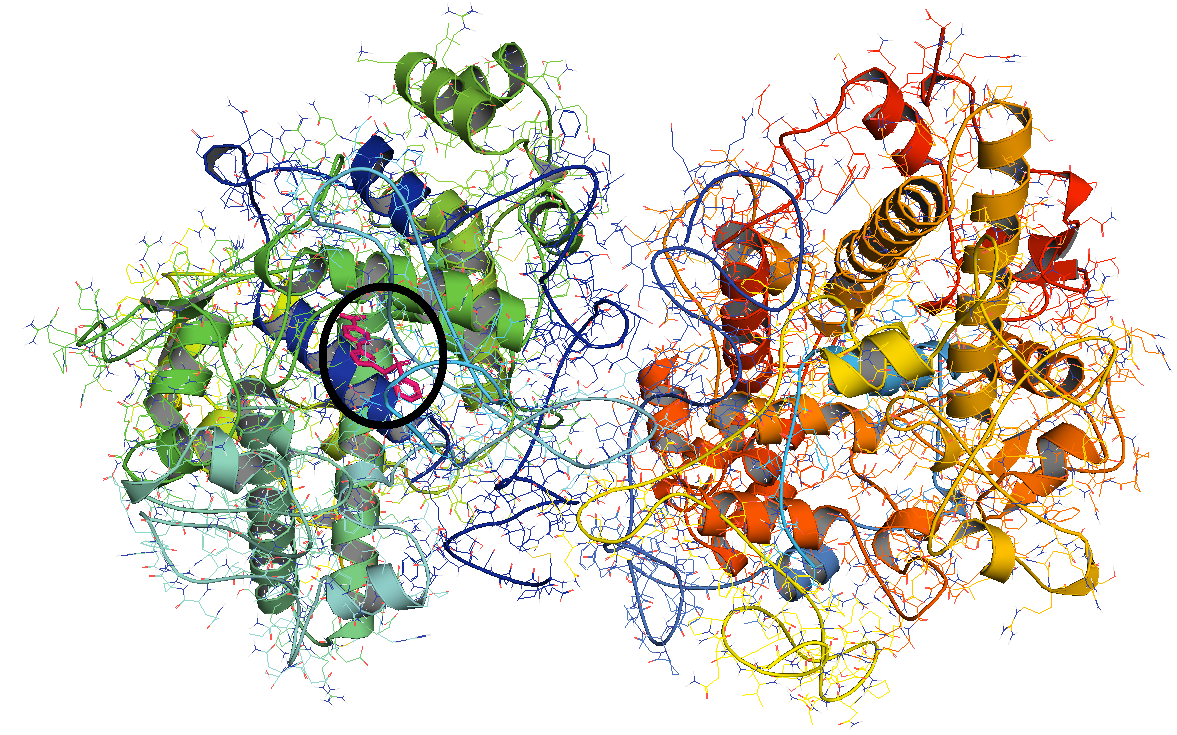

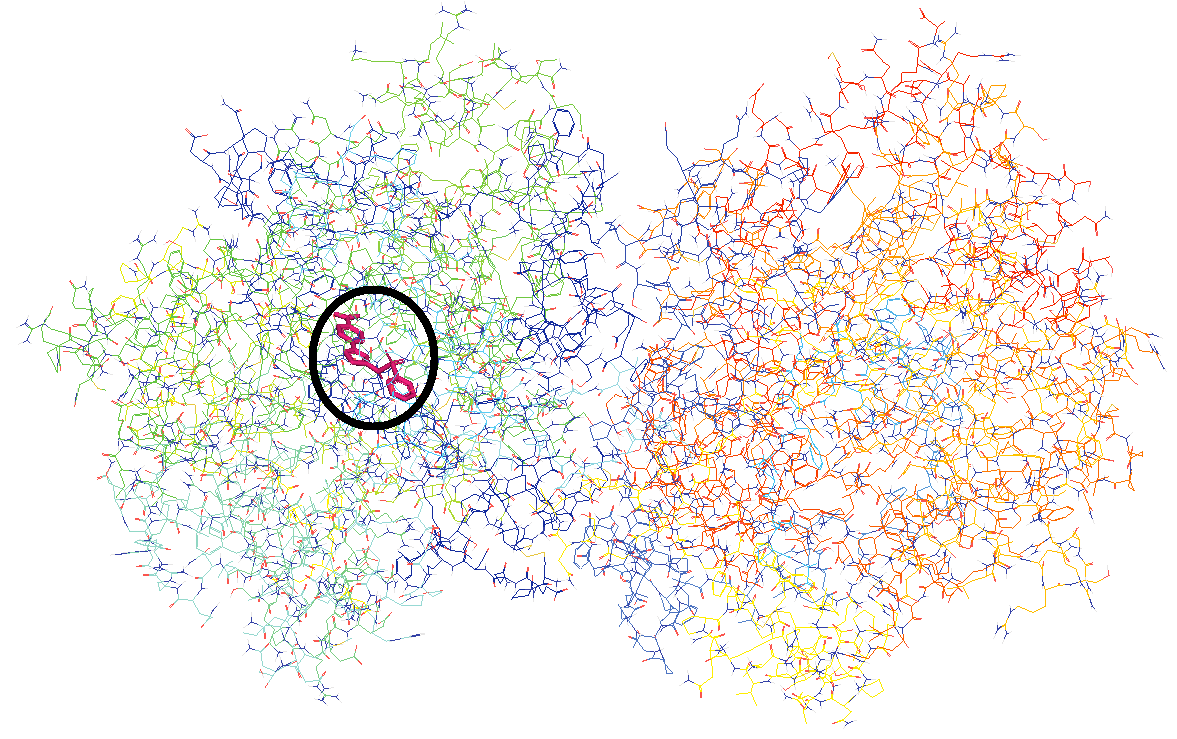


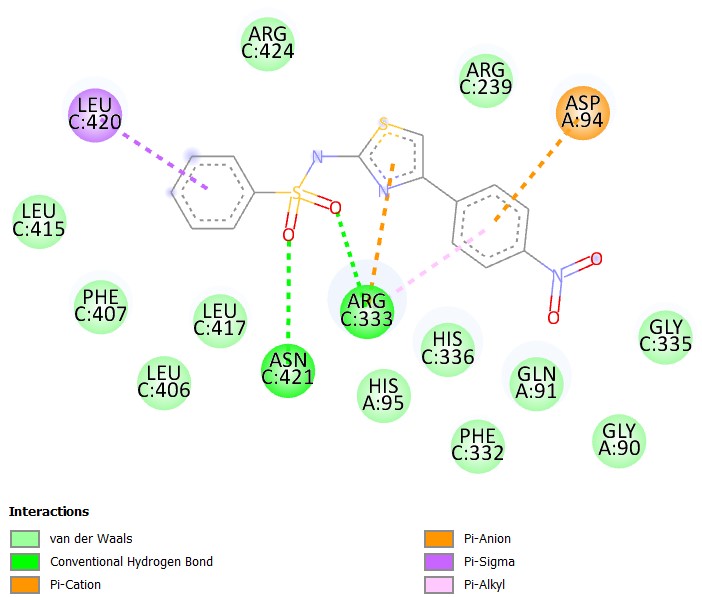

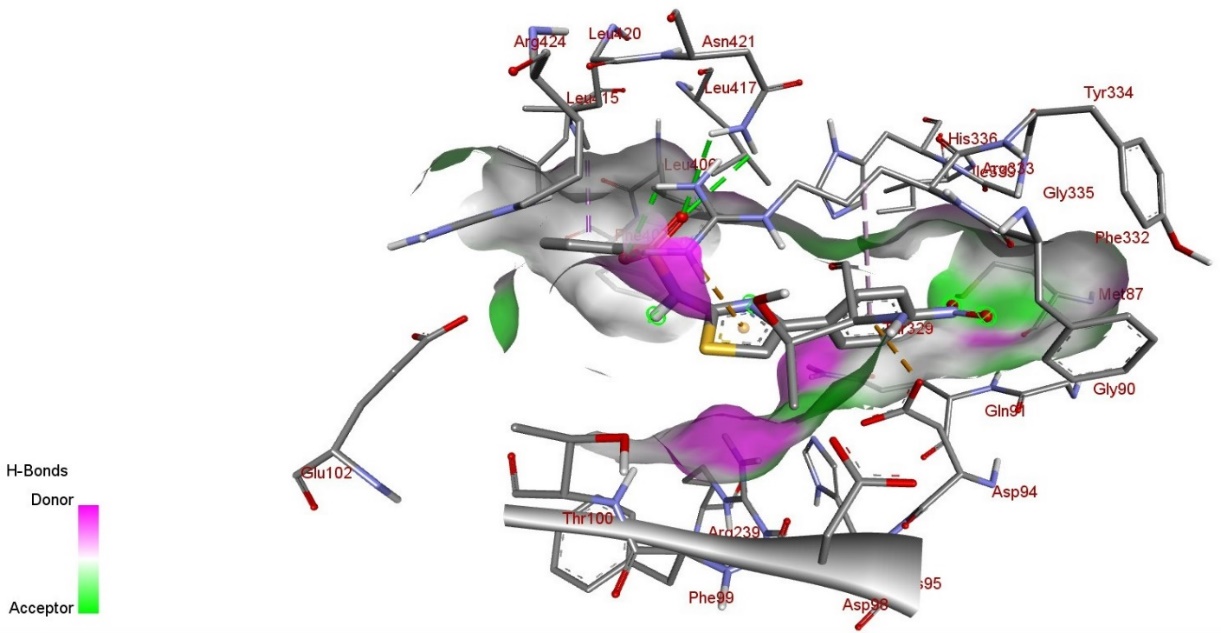


**Figure S16:** The 2D and 3D binding interactions of compound 11b against human myeloperoxidase (PDB ID: 1DNU). 3D Ribbon and line models show the binding pocket structure of human myeloperoxidase with compound 11b. Hydrogen bond between compounds and amino acids are shown as green dash lines, hydrophobic interactions are shown as pink/purple lines. Electrostatic interactions are shown as orange lines.


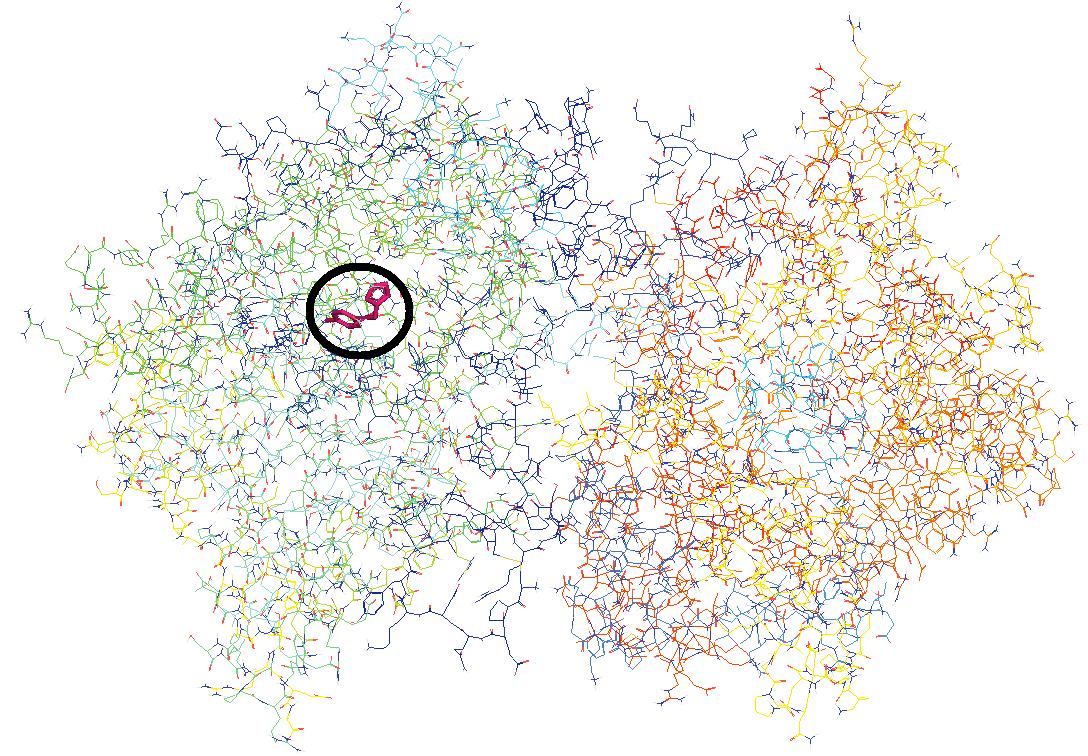

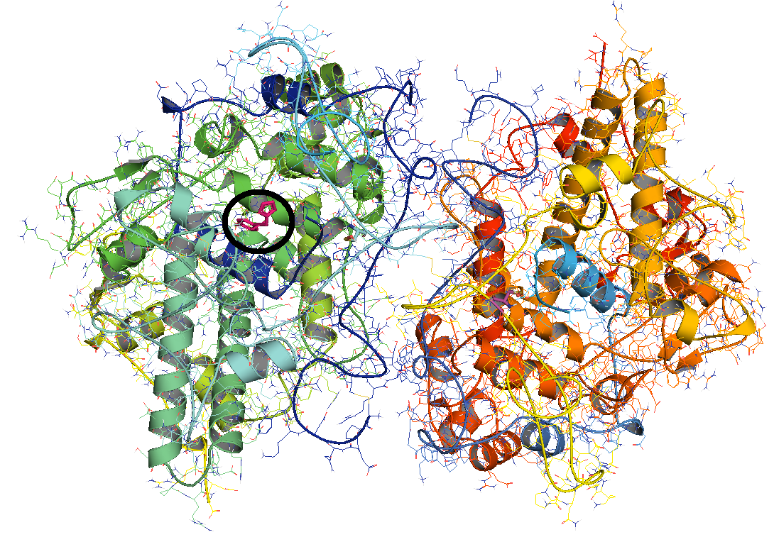
n as orange lines.


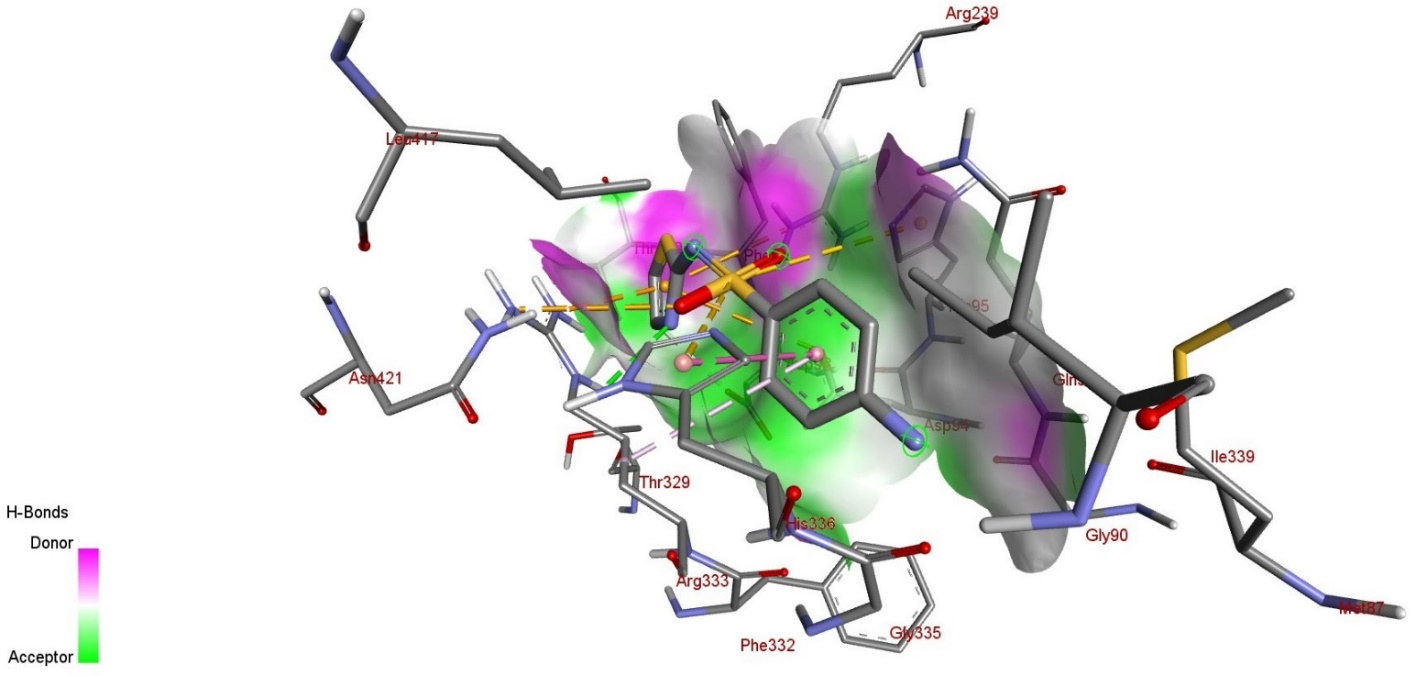


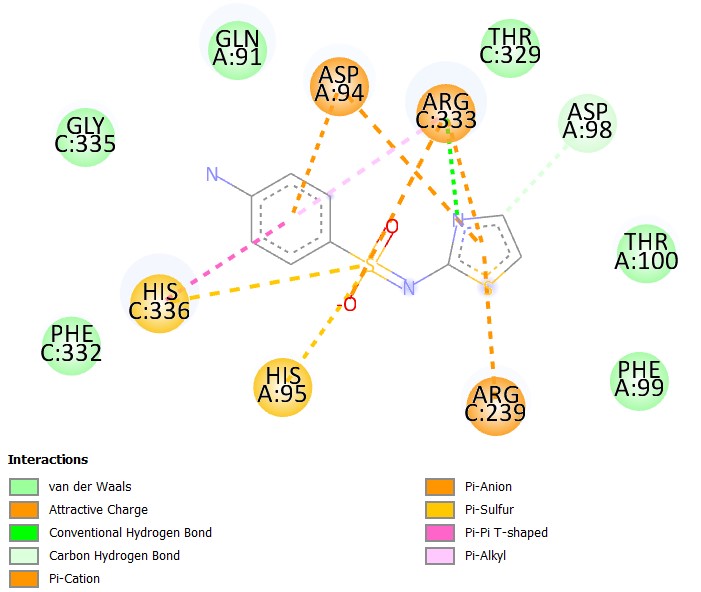


**Figure S17:** The 2D and 3D binding interactions of reference drug Sulfathiazole against human myeloperoxidase (PDB ID: 1DNU). 3D Ribbon and line models show the binding pocket structure of human myeloperoxidase with Sulfathiazole. Hydrogen bond between compounds and amino acids are shown as green dash lines, hydrophobic interactions are shown as pink lines. Electrostatic interactions are shown as orange lines.


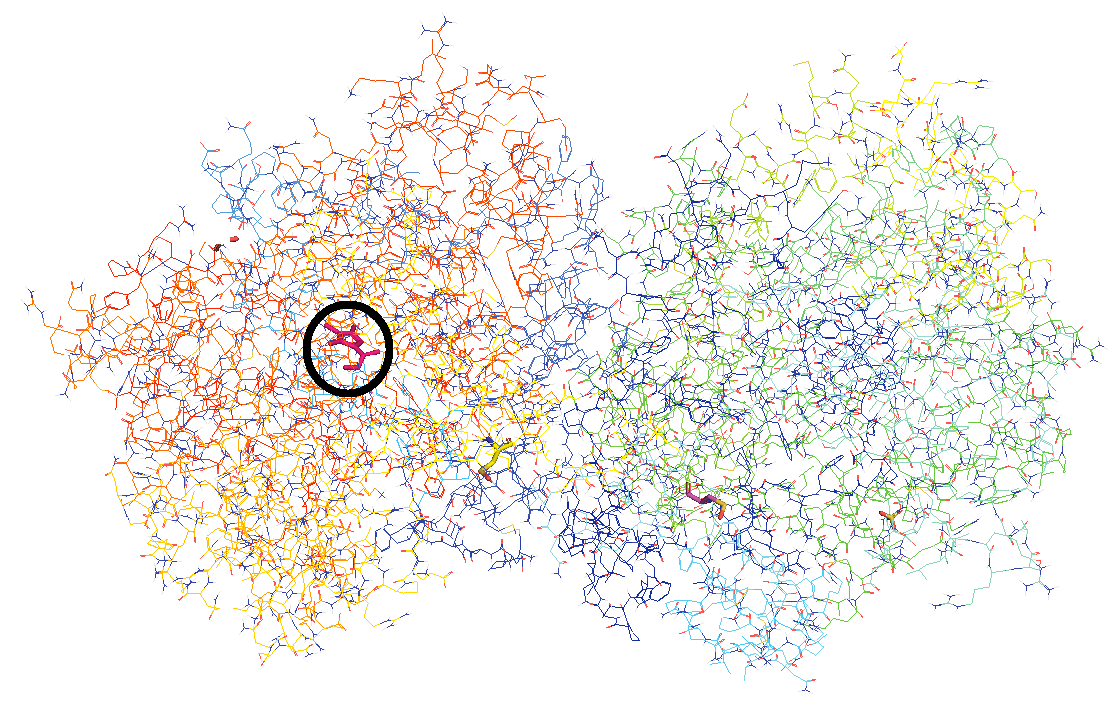

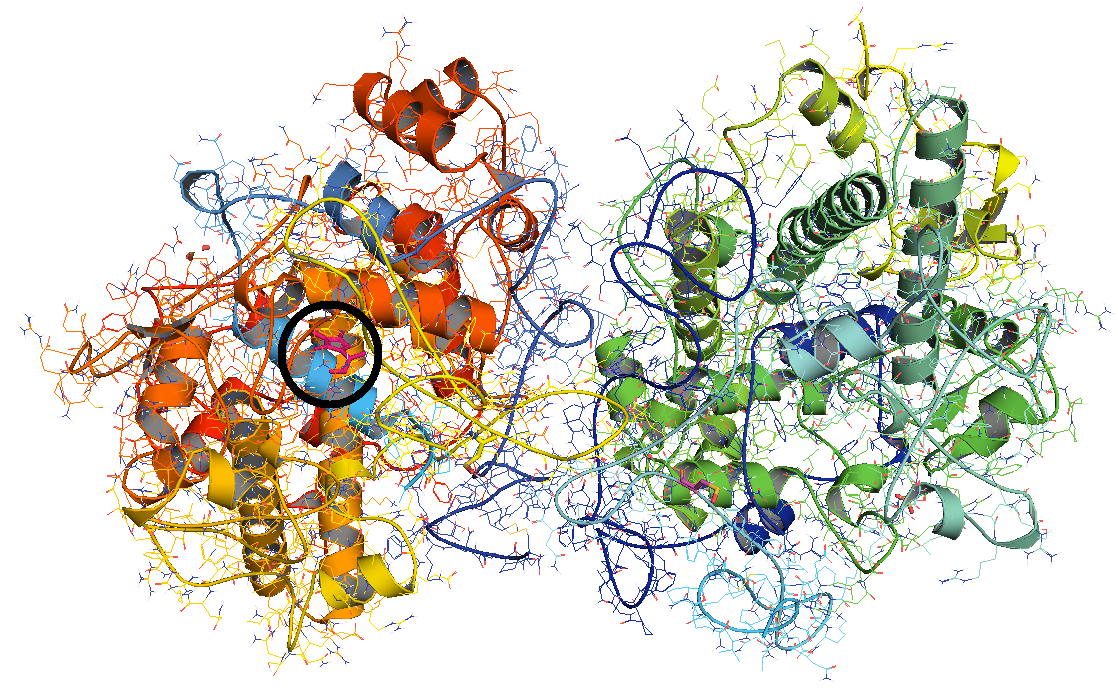


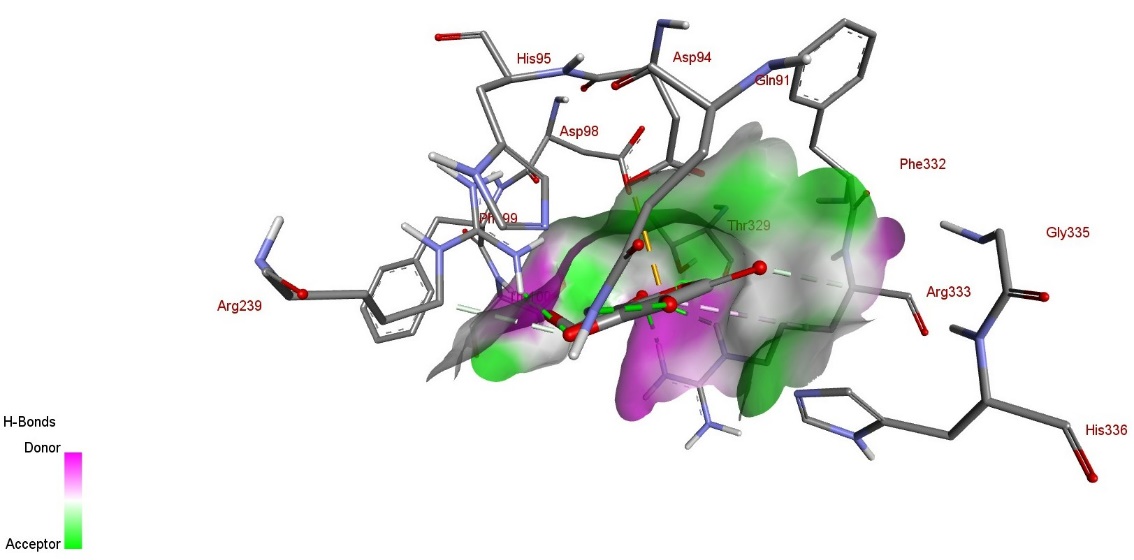

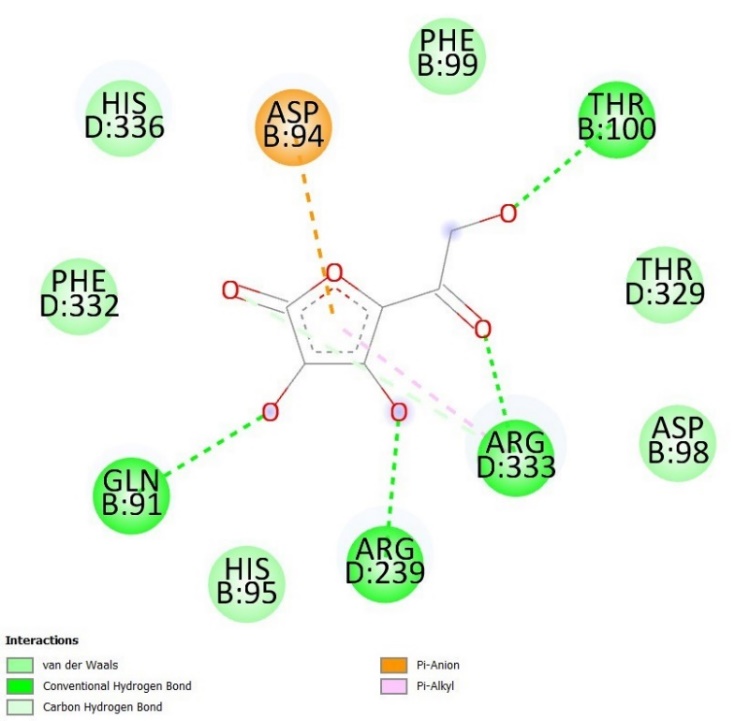


**Figure S18:** The 2D and 3D binding interactions of reference drug Ascorbic Acid against Human myeloperoxidase (PDB ID: 1DNU). 3D Ribbon and line models show the binding pocket structure of human myeloperoxidase with Ascorbic Acid. Hydrogen bond between compounds and amino acids are shown as green dash lines, Pi-anion interaction is shown as orange line. Hydrophobic interaction is shown as pink line.

**Figure S19:** Optimized Structures of Compounds (7, 11a-b) depicting force on nucleus.

**Figure S20:** Optimized Structures of Compounds (7, 11a-b) depicting Bond Length.

**Figure S21:** Optimized Structures of Compounds (7, 11a-b) depicting Mulliken Charges.

**Figure S22:** Optimized Structures of Compounds (7, 11a-b) depicting Molecular Electrostatic Potential Surface.

**Figure S23:** Optimized Structures of Compounds (7, 11a-b) depicting 2D Contour.

**Figure S24:** HOMO-LUMO Structures of Compounds (7, 11a-b).

**Optimized Parameters of synthesized compounds computed through Gaussian (R) 09 program**

**! Optimized Parameters Compound 7 !**

**! Bond Length (Angstroms) !**

**! Name Definition Value Derivative Info. !**

**--------------------------------------------------------------------------------**

! R1 R(1,2) 1.3693 -DE/DX = 0.0 !

! R2 R(1,5) 1.7434 -DE/DX = 0.0 !

! R3 R(1,16) 1.079 -DE/DX = 0.0 !

! R4 R(2,3) 1.3881 -DE/DX = 0.0 !

! R5 R(2,7) 1.4726 -DE/DX = 0.0001 !

! R6 R(3,4) 1.3002 -DE/DX = 0.0001 !

! R7 R(4,5) 1.7764 -DE/DX = 0.0 !

! R8 R(4,6) 1.3581 -DE/DX = 0.0 !

! R9 R(6,17) 1.0066 -DE/DX = 0.0 !

! R10 R(6,18) 1.0041 -DE/DX = 0.0 !

! R11 R(7,8) 1.4071 -DE/DX = 0.0 !

! R12 R(7,12) 1.4081 -DE/DX = 0.0 !

! R13 R(8,9) 1.3893 -DE/DX = 0.0 !

! R14 R(8,19) 1.083 -DE/DX = 0.0 !

! R15 R(9,10) 1.3935 -DE/DX = 0.0 !

! R16 R(9,20) 1.0826 -DE/DX = 0.0 !

! R17 R(10,11) 1.3954 -DE/DX = 0.0 !

! R18 R(10,13) 1.4662 -DE/DX = 0.0 !

! R19 R(11,12) 1.3872 -DE/DX = 0.0 !

! R20 R(11,21) 1.0825 -DE/DX = 0.0 !

! R21 R(12,22) 1.0846 -DE/DX = 0.0 !

! R22 R(13,14) 1.2325 -DE/DX = 0.0 !

! R23 R(13,15) 1.2322 -DE/DX = 0.0 !

**Mulliken charges: Compound 7**

1 C -0.350315

2 C 0.291890

3 N -0.515213

4 C 0.325941

5 S 0.215023

6 N -0.635532

7 C 0.096389

8 C -0.122718

9 C -0.094474

10 C 0.251124

11 C -0.101557

12 C -0.133005

13 N 0.381214

14 O -0.403680

15 O -0.402148

16 H 0.132522

17 H 0.293931

18 H 0.287168

19 H 0.122096

20 H 0.134990

21 H 0.135011

22 H 0.091345

**Sum of Mulliken charges = 0.00000**

**! Optimized Parameters Compound 11a !**

**! Bond Length (Angstroms) !**

**-------------------------- --------------------------**

**! Name Definition Value Derivative Info. !**

**--------------------------------------------------------------------------------**

! R1 R(1,2) 1.3701 -DE/DX = 0.0 !

! R2 R(1,5) 1.7394 -DE/DX = 0.0 !

! R3 R(1,26) 1.0794 -DE/DX = 0.0 !

! R4 R(2,3) 1.3869 -DE/DX = 0.0 !

! R5 R(2,7) 1.4728 -DE/DX = 0.0 !

! R6 R(3,4) 1.2903 -DE/DX = 0.0 !

! R7 R(4,5) 1.7743 -DE/DX = 0.0 !

! R8 R(4,6) 1.3923 -DE/DX = 0.0 !

! R9 R(6,16) 1.7481 -DE/DX = 0.0 !

! R10 R(6,27) 1.0157 -DE/DX = 0.0 !

! R11 R(7,8) 1.4068 -DE/DX = 0.0 !

! R12 R(7,12) 1.4072 -DE/DX = 0.0 !

! R13 R(8,9) 1.3898 -DE/DX = 0.0 !

! R14 R(8,28) 1.0836 -DE/DX = 0.0 !

! R15 R(9,10) 1.3934 -DE/DX = 0.0 !

! R16 R(9,29) 1.0826 -DE/DX = 0.0 !

! R17 R(10,11) 1.395 -DE/DX = 0.0 !

! R18 R(10,13) 1.4676 -DE/DX = 0.0 !

! R19 R(11,12) 1.3876 -DE/DX = 0.0 !

! R20 R(11,30) 1.0824 -DE/DX = 0.0 !

! R21 R(12,31) 1.0849 -DE/DX = 0.0 !

! R22 R(13,14) 1.2324 -DE/DX = 0.0 !

! R23 R(13,15) 1.2315 -DE/DX = 0.0 !

! R24 R(16,17) 1.7891 -DE/DX = 0.0 !

! R25 R(16,18) 1.4574 -DE/DX = 0.0 !

! R26 R(16,19) 1.4636 -DE/DX = 0.0 !

! R27 R(17,20) 1.3975 -DE/DX = 0.0 !

! R28 R(17,24) 1.3947 -DE/DX = 0.0 !

! R29 R(20,21) 1.3908 -DE/DX = 0.0 !

! R30 R(20,32) 1.0845 -DE/DX = 0.0 !

! R31 R(21,22) 1.4046 -DE/DX = 0.0 !

! R32 R(21,33) 1.0865 -DE/DX = 0.0 !

! R33 R(22,23) 1.4011 -DE/DX = 0.0 !

! R34 R(22,25) 1.5088 -DE/DX = 0.0 !

! R35 R(23,24) 1.3944 -DE/DX = 0.0 !

! R36 R(23,34) 1.0863 -DE/DX = 0.0 !

! R37 R(24,35) 1.0845 -DE/DX = 0.0 !

! R38 R(25,36) 1.0941 -DE/DX = 0.0 !

! R39 R(25,37) 1.0967 -DE/DX = 0.0 !

! R40 R(25,38) 1.093 -DE/DX = 0.0 !

**Mulliken charges: Compound 11a**

1 C -0.346901

2 C 0.294910

3 N -0.472968

4 C 0.304644

5 S 0.238800

6 N -0.691577

7 C 0.095845

8 C -0.126696

9 C -0.095454

10 C 0.252446

11 C -0.101490

12 C -0.132573

13 N 0.382477

14 O -0.403195

15 O -0.399146

16 S 1.254438

17 C -0.237698

18 O -0.508662

19 O -0.500074

20 C -0.059711

21 C -0.116181

22 C 0.129709

23 C -0.117833

24 C -0.077000

25 C -0.383368

26 H 0.139445

27 H 0.294214

28 H 0.148657

29 H 0.139705

30 H 0.135449

31 H 0.090565

32 H 0.139315

33 H 0.100099

34 H 0.099383

35 H 0.140493

36 H 0.127064

37 H 0.141292

38 H 0.121578

**Sum of Mulliken charges = 0.00000**

**! Optimized Parameters Compound 11b !**

**! Bond Length (Angstroms) !**

**-------------------------- --------------------------**

**! Name Definition Value Derivative Info. !**

**--------------------------------------------------------------------------------**

! R1 R(1,2) 1.3701 -DE/DX = 0.0 !

! R2 R(1,5) 1.7394 -DE/DX = 0.0 !

! R3 R(1,25) 1.0794 -DE/DX = 0.0 !

! R4 R(2,3) 1.3871 -DE/DX = 0.0 !

! R5 R(2,7) 1.4728 -DE/DX = 0.0 !

! R6 R(3,4) 1.2901 -DE/DX = 0.0 !

! R7 R(4,5) 1.7739 -DE/DX = 0.0 !

! R8 R(4,6) 1.3932 -DE/DX = 0.0 !

! R9 R(6,16) 1.7458 -DE/DX = 0.0 !

! R10 R(6,26) 1.0159 -DE/DX = 0.0 !

! R11 R(7,8) 1.4068 -DE/DX = 0.0 !

! R12 R(7,12) 1.4071 -DE/DX = 0.0 !

! R13 R(8,9) 1.3898 -DE/DX = 0.0 !

! R14 R(8,27) 1.0835 -DE/DX = 0.0 !

! R15 R(9,10) 1.3934 -DE/DX = 0.0 !

! R16 R(9,28) 1.0826 -DE/DX = 0.0 !

! R17 R(10,11) 1.395 -DE/DX = 0.0 !

! R18 R(10,13) 1.4678 -DE/DX = 0.0 !

! R19 R(11,12) 1.3876 -DE/DX = 0.0 !

! R20 R(11,29) 1.0824 -DE/DX = 0.0 !

! R21 R(12,30) 1.0849 -DE/DX = 0.0 !

! R22 R(13,14) 1.2324 -DE/DX = 0.0 !

! R23 R(13,15) 1.2315 -DE/DX = 0.0 !

! R24 R(16,17) 1.7934 -DE/DX = 0.0 !

! R25 R(16,18) 1.4634 -DE/DX = 0.0 !

! R26 R(16,19) 1.4572 -DE/DX = 0.0 !

! R27 R(17,20) 1.3971 -DE/DX = 0.0 !

! R28 R(17,24) 1.3956 -DE/DX = 0.0 !

! R29 R(20,21) 1.3934 -DE/DX = 0.0 !

! R30 R(20,31) 1.0844 -DE/DX = 0.0 !

! R31 R(21,22) 1.3979 -DE/DX = 0.0 !

! R32 R(21,32) 1.0854 -DE/DX = 0.0 !

! R33 R(22,23) 1.396 -DE/DX = 0.0 !

! R34 R(22,33) 1.0858 -DE/DX = 0.0 !

! R35 R(23,24) 1.3956 -DE/DX = 0.0 !

! R36 R(23,34) 1.0854 -DE/DX = 0.0 !

! R37 R(24,35) 1.0845 -DE/DX = 0.0 !

**Mulliken charges: Compound 11b**

1 C -0.346720

2 C 0.294547

3 N -0.472638

4 C 0.304295

5 S 0.240839

6 N -0.692522

7 C 0.096032

8 C -0.126714

9 C -0.095375

10 C 0.252451

11 C -0.101380

12 C -0.132569

13 N 0.382710

14 O -0.402891

15 O -0.398870

16 S 1.256445

17 C -0.231541

18 O -0.498607

19 O -0.508089

20 C -0.059866

21 C -0.085687

22 C -0.072150

23 C -0.084248

24 C -0.078634

25 H 0.140021

26 H 0.295086

27 H 0.148443

28 H 0.139951

29 H 0.135766

30 H 0.090781

31 H 0.141211

32 H 0.110158

33 H 0.107827

34 H 0.109411

35 H 0.142528

**Sum of Mulliken charges = 0.00000**
